# Comparative Magnitude and Persistence of Humoral SARS-CoV-2 Vaccination Responses in the Adult Population in Germany

**DOI:** 10.3389/fimmu.2022.828053

**Published:** 2022-02-16

**Authors:** Alex Dulovic, Barbora Kessel, Manuela Harries, Matthias Becker, Julia Ortmann, Johanna Griesbaum, Jennifer Jüngling, Daniel Junker, Pilar Hernandez, Daniela Gornyk, Stephan Glöckner, Vanessa Melhorn, Stefanie Castell, Jana-Kristin Heise, Yvonne Kemmling, Torsten Tonn, Kerstin Frank, Thomas Illig, Norman Klopp, Neha Warikoo, Angelika Rath, Christina Suckel, Anne Ulrike Marzian, Nicole Grupe, Philipp D. Kaiser, Bjoern Traenkle, Ulrich Rothbauer, Tobias Kerrinnes, Gérard Krause, Berit Lange, Nicole Schneiderhan-Marra, Monika Strengert

**Affiliations:** ^1^NMI Natural and Medical Sciences Institute at the University of Tübingen, Reutlingen, Germany; ^2^Department of Epidemiology, Helmholtz Centre for Infection Research, Braunschweig, Germany; ^3^German Red Cross Blood Donation Service North East, Dresden, Germany; ^4^Hannover Unified Biobank, Hannover Medical School, Hannover, Germany; ^5^Pharmaceutical Biotechnology, Department of Pharmacy and Biochemistry, University of Tübingen, Tübingen, Germany; ^6^Department of RNA-Biology of Bacterial Infections, Helmholtz Institute for RNA-Based Infection Research, Würzburg, Germany; ^7^TWINCORE, Centre for Experimental and Clinical Infection Research, a Joint Venture of the Hannover Medical School and the Helmholtz Centre for Infection Research, Hannover, Germany; ^8^German Centre for Infection Research (DZIF), Partner Site Hannover-Braunschweig, Braunschweig, Germany

**Keywords:** SARS-CoV-2, mRNA vaccines, vector-based vaccines, variants of concern, protective immunity, population-based study, longitudinal study, antibody persistence

## Abstract

Recent increases in SARS-CoV-2 infections have led to questions about duration and quality of vaccine-induced immune protection. While numerous studies have been published on immune responses triggered by vaccination, these often focus on studying the impact of one or two immunisation schemes within subpopulations such as immunocompromised individuals or healthcare workers. To provide information on the duration and quality of vaccine-induced immune responses against SARS-CoV-2, we analyzed antibody titres against various SARS-CoV-2 antigens and ACE2 binding inhibition against SARS-CoV-2 wild-type and variants of concern in samples from a large German population-based seroprevalence study (MuSPAD) who had received all currently available immunisation schemes. We found that homologous mRNA-based or heterologous prime-boost vaccination produced significantly higher antibody responses than vector-based homologous vaccination. Ad26.CoV2S.2 performance was particularly concerning with reduced titres and 91.7% of samples classified as non-responsive for ACE2 binding inhibition, suggesting that recipients require a booster mRNA vaccination. While mRNA vaccination induced a higher ratio of RBD- and S1-targeting antibodies, vector-based vaccines resulted in an increased proportion of S2-targeting antibodies. Given the role of RBD- and S1-specific antibodies in neutralizing SARS-CoV-2, their relative over-representation after mRNA vaccination may explain why these vaccines have increased efficacy compared to vector-based formulations. Previously infected individuals had a robust immune response once vaccinated, regardless of which vaccine they received, which could aid future dose allocation should shortages arise for certain manufacturers. Overall, both titres and ACE2 binding inhibition peaked approximately 28 days post-second vaccination and then decreased.

## Introduction

In response to the global SARS-CoV-2 pandemic, multiple vaccines have been developed, tested and licensed for use within record time (1–4). As vaccination coverage became more widespread at the beginning of 2021, countries experienced a reduction in SARS-CoV-2 infections (5, 6), although case numbers have again begun to increase in recent months due to spread among and by unvaccinated individuals (7) as well as longevity-related reductions in vaccine protection (8–11). Although a measurable correlate of protection that either prevents SARS-CoV-2 infection or limits COVID-19 disease progression is not yet defined, sufficient levels of neutralizing antibodies are assumed to be a key element (12, 13). As in most other countries, the German national vaccination strategy (until June 7^th^ 2021) was based on prioritisation by occupation, underlying medical conditions or advanced age. Currently, 56.8 million German residents are reported to be completely vaccinated (68.3% coverage), with a further 2.4 million having so far received one dose. The majority of doses administered based on delivery numbers in Germany are BNT162b2 from Pfizer (77.0%), followed by Astra Zeneca´s AZD1222 (11.3%), Moderna’s mRNA-1273 (8.7%) and Janssen’s single-shot Ad26.CoV2.S (3.0%; impfdashboard.de and rki.de as of November 25^th^ 2021). However, based on a lack of efficacy data from phase III clinical trials, the German Standing Committee on Vaccination (STIKO) recommended AZD1222 only for use in those below the age of 60. Following reports of moderate to severe thrombocytopenia and atypical thrombosis cases after AZD1222 vaccination in spring 2021 (14–16), temporary suspensions and eligibility restrictions were not only enacted in Germany (on March 15^th^ 2021) but in 12 other EU member states (17). Administration of AZD1222 was resumed by the 1^st^ of April 2021 in Germany, however only for those above the age of 60 or after an individual risk analysis. Individuals who had received a first dose of AZD1222 and were below the age of 60 were instead offered a mRNA-based vaccine as second dose which resulted in a heterologous prime-boost vaccination scheme (18). Although these “mix and match” approaches were not covered by the initial licensing terms, it has by now been shown that they result in a more robust humoral and cell-mediated immune response compared to the homologous AZD1222 immunisation (19, 20). While multiple studies have so far investigated vaccine-induced responses, predominantly in at-risk groups such as dialysis or transplant recipients (21, 22), groups with increased exposure risk such as health care workers (23–25) or as part of the initial clinical efficacy trials which in general enroll healthier than average populations (26), we report immunological vaccination response data from the general adult population. By using samples from a population-based seroprevalence study (MuSPAD), which assessed SARS-CoV-2 seroprevalence from July 2020 to August 2021 in eight regions in Germany (27), we examined the dynamics of vaccine-induced humoral responses using MULTICOV-AB (28) and an ACE2-RBD competition assay (29) to analyze ACE2 binding inhibition.

## Material and Methods

### MuSPAD Study Recruitment

Vaccination responses were analyzed in participants of the multi-local and serial cross-sectional prevalence study on antibodies against SARS-CoV-2 in Germany (MuSPAD) study, a nationwide population-based SARS-CoV-2 seroprevalence study (27) from July 2020 to August 2021. The study was approved by the Ethics Committee of the Hannover Medical School (9086_BO_S_2020). MuSPAD participants were recruited by age- and gender-stratified random sampling based on records from the respective local residents’ registration offices. Study locations in eight regions across Germany were selected in spring 2020 based on differing epidemic activity at that time. In addition to the successive cross-sectional study design, certain study locations were sampled longitudinally within a 3-4 month interval. At the study center, following written informed consent, all eligible participants (>18 years) were subject to a standardised computer-based interview using the digital eResearch system PIA (Prospective Monitoring and Management-App) to gather basic sociodemographic data, information on pre-existing medical conditions including a previously confirmed SARS-CoV-2 infection or a SARS-CoV-2 vaccination, once it became available in Germany in late December 2020. Information about SARS-CoV-2 infections or vaccinations are self-reported. After serum was obtained by venipuncture from a serum gel S-Monovette (Sarstedt), samples were aliquoted in Matrix 2D Barcoded Screw Top Tubes (Thermo Scientific) at the Institute of Transfusion Medicine and Immunohematology and frozen at -20°C before being transported on dry ice to the Hannover Unified Biobank (Germany). After registration and quality control, one serum aliquot was shipped to the Natural and Medical Sciences Institute (Reutlingen, Germany) where they were stored at -80°C until analysis.

### Study Design and Eligibility

Our study contains a total of 1821 samples from 1731 MuSPAD participants which were divided into three subgroups to examine different aspects of the vaccine-induced humoral response. Based on our inclusion criteria, individual samples can be part of several subgroups.

1. Individuals who received a homologous or heterologous complete two-dose vaccination with AZD1222, BNT162b2 and mRNA-1273 or the one-dose vaccine Ad26.CoV2.S with a blood sample taken at least 7 days but no more than 65 days post the last vaccination (hereon referred to as “mix and match sample cohort”).
2. Individuals who donated one blood sample following a two-dose homologous vaccination with BNT162b2 or mRNA-1273 within the defined time frames of day 5 to 12, day 26 to 30, day 54 to 58, day 94 to 103, day 129 to 146 or day 176 to 203 after the second dose to monitor antibody kinetics (hereon referred to as “time point sample cohort”).
3. Individuals with paired blood samples taken at two separate successive time points where the first sample had to be taken a minimum of seven days after the second homologous dose of BNT162b2 (hereon referred to as “longitudinal sample cohort”).

All samples originated from the following locations where the MuSPAD study had previously been scheduled to take place and were collected from January to August 2021: Aachen (Städteregion), Magdeburg (Stadtkreis), Osnabrück (Stadt- und Landkreis), Chemnitz (Stadtkreis) or Landkreis Vorpommern-Greifswald. A flow chart to illustrate sample selection form the entire MuSPAD cohort can be found in **Supplementary Figure 1**. Basic sociodemographic information and details of comorbidities (hypertension, cardiovascular disease, diabetes, lung disease, immunosuppression, cancer) for each group are provided in more detail in **Table 1** and **Supplementary Table 1**. Apart from the homologous BNT162b2 samples which are part of our mix and match sample cohort, the maximum available sample number meeting the specified criteria in groups 1-3 was used. For the homologous BNT162b2 vaccination samples within our mix and match sample cohort, we applied a random selection from the entire available sample pool of BNT162b2 vaccinees who took part in the MuSPAD study to select 771 sera. Individuals with a previous SARS-CoV-2 infection either defined by a positive SARS-CoV-2 PCR or antigen test result, or a MULTICOV-AB nucleocapsid IgG normalisation ratio above 1 are listed separately (hereon referred to as “recovered”) within the mix and match sample cohort. Additional sample eligibility criteria were having a complete vaccination record (manufacturer and vaccination dates) and information on age and gender as part of the participant’s metadata.

**Table 1:** Demographics of study population (n. a., not applicable; NA, not available).

Sample cohort (n)	SARS-CoV-2 infection status* (n)	Vaccine (n)	Mean ΔT (SD) in days post-vaccination	Mean ΔT (SD) in days between doses	Age (y), median (IQR)	Female (n, %)	Comorbidities (min. 1/person) (n, %)	Number of comorbidities (mean, SD)
**Mix and match (1470)**	+ (70)	M/M (13)	34.7 (16.3)	42.5 (27.9)	59 (11)	8 (61.5)	5 (38.5)	0.5 (0.8)
		P/P (33)	31.5 (13.5)	25.3 (9.6)	66 (29)	25 (75.8)	13 (39.4)	0.6 (0.9)
		A/A (12)	35.3 (13.5)	70.3 (19.8)	69 (7)	7 (58.3)	10 (83.3)	1.3 (0.9)
		A/M (1)**	17.0 (0.0)	66.0 (0.0)	age group 66-79	0 (0.0)	1 (100.0)	1.0 (0.0)
		A/P (6)	47.8 (14.5)	72.0 (11.6)	57 (25)	3 (50.0)	4 (66.7)	0.8 (0.7)
		J (5)	42.2 (18.0)	n. a.	40 (15)	3 (60.0)	3 (60.0)	0.6 (0.5)
	- (1400)	M/M (272)	36.5 (16.5)	31.2 (7.1)	56 (26)	162 (59.6)	108 (39.7)	0.6 (0.8)
		P/P (738)	34.7 (17.1)	27.9 (9.7)	59 (26)	456 (61.8)	438 (4 NA; 46.1)	0.7 (1.0)
		A/A (228)	37.3 (13.8)	73.6 (10.9)	66 (10)	122 (53.5)	114 (50.0)	0.7 (0.9)
		A/M (24)	20.8 (7.8)	69.6 (16.9)	68 (5)	15 (62.5)	14 (58.3)	0.8 (0.8)
		A/P (114)	31.6 (17.4)	72.2 (14.8)	59 (20)	64 (56.1)	55 (48.3)	0.8 (1.0)
		J (24)	49.8 (10.8)	n. a.	62 (14)	15 (62.5)	11 (45.8)	0.8 (1.2)
**Time points (597)**	–	P/P (107)	6.9 (1.4)	34.7 (13.5)	64 (26)	57 (53.3)	61 (57.0)	0.8 (0.9)
		M/M (40)	6.7 (1.3)	39.7 (11.2)	58 (27)	20 (50.0)	15 (37.5)	0.5 (0.8)
		P/P (103)	27.9 (1.4)	28.2 (9.9)	52 (42)	60 (58.3)	42 (1 NA; 41.2)	0.8 (1.1)
		M/M (8)	28.1 (1.4)	30.3 (4.7)	67 (39)	5 (62.5)	3 (37.5)	0.5 (0.7)
		P/P (92)	55.9 (1.5)	29.1 (9.6)	61 (20)	60 (65.2)	45 (48.9)	0. 8 (1.0)
		M/M (22)	55.5 (1.2)	28.0 (0.3)	57 (15)	17 (77.3)	5 (22.7)	0.3 (0.6)
		P/P (139)	97.7 (2.6)	22.3 (4.0)	60 (22)	87 (62.6)	72 (51.8)	0.8 (1.0)
		M/M (7)	98.1 (2.6)	28.9 (2.3)	64 (8)	5 (71.4)	3 (42.9)	0.9 (1.1)
		P/P (38)	138.0 (3.8)	21.0 (2.2)	80 (24)	25 (68.8)	24 (63.1)	1.2 (1.2)
		M/M (5)	141.0 (5.1)	29.2 (1.6)	83 (4)	2 (40.0)	3 (60.0)	1.2 (1.2)
		P/P (36)	189.0 (5.6)	21.7 (1.4)	50 (13)	30 (83.3)	11 (30.6)	0.4 (0.6)
		M/M (0)	n. a	n. a.	n. a.	n. a.	n. a.	n. a.
**Longitudinal (180)**	–	P/P T1 (90)	27.9 (14.8)	21.3 (1.2)	58 (34)	65 (72.2)	45 (2 NA; 51.1)	0.8 (1.0)
		P/P T2 (90)	166.4 (19.4)		58 (33)	65 (72.2)	48 (53.3)	0.7 (1.0)

Different vaccines and combinations are abbreviated as follows: M/M (two-dose mRNA-1273), P/P (two-dose BNT162b2), A/A (two-dose AZD1222), A/M (first dose AZD1222, second dose mRNA-1273), A/P (first dose AZD1222, second dose BNT162b2) and J (one-dose Ad26.CoV2.S). The time points sample cohort contains only homologous BNT162b2 and mRNA-1273 samples. The longitudinal sample cohort contains only paired homologous BNT162b2 taken at time 1 (T1) or 2 (T2).

*Based on self-reported positive PCR/antigen test result at study center visit and/or MULTICOV-AB nucleocapsid IgG S/CO ratio above 1; **only age group reported as n=1.

### MULTICOV-AB

Vaccine-induced humoral responses were analyzed using MULTICOV-AB (28), a previously published semi-quantitative multiplex immunoassay that includes both antigens of SARS-CoV-2 (e.g. Spike, Receptor Binding Domain (RBD), S1 domain, S2 domain and nucleocapsid) and the endemic coronaviruses (OC43, HKU1, NL63 and 229E). While samples were processed using an automated platform on a Beckman Coulter i7 pipetting robot as previously described (30) with minor modifications, all sample and reagent dilutions were already established and verified as part of the initial MULTICOV-AB technical assay validation process which is detailed in (28). Briefly, samples were thawed at room temperature, vortexed and then centrifuged at 2000 g for 3 mins to pellet any cell debris within the sample. Samples were then opened using a LabElite DeCapper SL (Hamilton Company). Opened sample matrix racks were then loaded into the pipetting robot, where the sample was diluted 1:200 in assay buffer, before being combined in a 384-well plate and mixed 1:1 with 1x bead mix (see **Supplementary Table 2** for antigen panel), resulting in a final dilution of 1:400. Samples were then incubated in a Thermomixer (Eppendorf) for 2 h at 1400 rpm, 20°C, in darkness. Following this initial incubation, samples were washed to remove unbound antibodies using an automated magnetic plate washer (Biotek). Bound IgG was detected by adding R-phycoerythrin labelled goat-anti-human IgG (3 µg/mL; #109-116-098, Jackson Immunoresearch Labs) and incubating for a further 45 mins at 1400 rpm, 20°C, in darkness. Following a further washing step, beads were resuspended in 100 µl of wash buffer, shaken for 1 min at 1400 rpm and then measured once on a FLEXMAP 3D instrument (Luminex Corporation) using the following settings: Timeout 100 sec, Gate 7500-15000, Reporter Gain: Standard PMT, 40 events. To ensure reproducibility, 3 quality control (QC) samples were included in octuplicate per plate. Additionally, each plate had to pass 3 QC criteria to be considered as valid run: first, throughout acquisition each sample had to reach a minimum bead count of 35 per bead ID, second median fluorescence intensity (MFI) values of sample and signal system control beads and third plate-by-plate QC sample controls had to be within normal range. Beads coupled with human IgG and goat-anti-human IgG were utilized to control for sample and signal system addition. Any sample that failed QC was remeasured for MULTICOV-AB and the ACE2-RBD competition assay (30/1821). Raw MFI values were normalised to a QC sample for all antigens as in (24, 31). A Signal to Cutoff ratio (S/CO) of 1 or above for both the trimeric Spike and RBD antigen was defined as reactive for SARS-CoV-2 Spike-specific IgG.

### ACE2-RBD Competition Assay

To enable high-throughput screening of ACE2-RBD binding inhibition in the presence of sera, a previously established ACE2-RBD competition assay (29) was automated on a Beckmann Coulter i7 pipetting robot with minor modifications. 1:20 previously diluted samples from MULTICOV-AB were diluted 1:200 in ACE2 buffer (29) containing 150 ng/mL biotinylated ACE2. Samples were then mixed 1:1 with 1x VoC (Variant of Concern) bead mix containing RBDs of SARS-CoV-2 wild-type and the Alpha, Beta, Gamma, Delta VoCs (**Supplementary Table 3**), resulting in a final dilution of 1:400. Samples were then incubated in a Thermomixer for 2 h at 1400 rpm, 20°C, in darkness. Following this initial incubation, samples were washed to remove unbound ACE2 using an automated magnetic plate washer. ACE2 was detected using R-phycoerythrin labelled streptavidin (2 µg/mL, #SAPE-001, Moss) by incubating the bead-sample mix for a further 45 mins at 1400 rpm, 20°C, in darkness. Following a further washing step, beads were resuspended in 100 µl of wash buffer, shaken for 1 min at 1400 rpm and then measured once on a FLEXMAP 3D instrument using the following settings: Timeout 100 sec, Gate 7500-15000, Reporter Gain: Standard PMT, 40 events. As controls, 12 blank wells, 10 wells with 150 ng/mL ACE2 alone and 10 wells with an ACE2 QC sample were included. ACE2 binding inhibition was calculated as percentage ACE2 inhibition as in (29) with 100% indicating maximum ACE2 binding inhibition and 0% no ACE2 binding inhibition. Samples with an ACE2 binding inhibition less than 20% are classified as non-responders (29).

### Data Analysis and Statistics

Initial results collation and matching to metadata was done in Excel 2016 and R 4.1.0 (32).

For pair-wise comparisons of titres and ACE2 binding inhibition between vaccination schemes within our mix and match sample cohort, we used a two-sided generalized Wilcoxon test also referred to as Brunner-Munzel test (33) with a significance level of 0.05 as part of the lawstat package (34). In each comparison of two vaccination schemes, the test assesses if a titre (or ACE2 binding inhibition) tends to larger (smaller) values under one vaccination scheme in comparison to the other. Where indicated, we adjusted for multiple testing by using the Bonferroni-Holm’s procedure (35) to control the family-wise error rate to be at most 0.05.

To investigate the impact of age, sex, comorbidities and time post-vaccination on the ACE2 binding inhibition between the different vaccination schemes, we used a normal linear mixed model for logit-transformed ACE2 binding inhibition. Negative measurement values were replaced by 0.001 to enable the transformation. The model included additive effects of age, sex, time post-vaccination (peak response period: 7-27 days vs plateau response period: 28-65 days) and comorbidities [cardiovascular disease, hypertension, diabetes, lung disease and cancer/immunosuppression (which were combined to a binary indicator based on low sample numbers)]. The model further included a random effect defined by the variable “plate number” to account for dependencies due to the measurement procedure, and allowed for heteroscedastic variances for younger (<=70) and older (>70) ages and vaccination types. REML estimation was implemented using the lme function [nlme library (36)]. Statistical testing was based on the asymptotic normality of the estimates. As part of a sensitivity analysis, we extended the model with interaction terms between each confounder and the time post-vaccination, allowing for possibly differing effects in the peak (7-27 days) and plateau (28-65 days) period after the last vaccination. Since the effects of the considered covariates were allowed to differ between the individual vaccination schemes, we analyzed only four vaccination schemes (BNT162b2-BNT162b2, mRNA-1273-mRNA-1273, AZD1222-BNT162b2, AZD1222-AZD1222) with a reasonable sample size in the mix and match study cohort, while we excluded immunisation with AZD1222-mRNA-1273 and Ad26.CoV2.S due to the low sample number of 24 per scheme. Additionally, four individuals with a BNT162b2-BNT162b2 vaccination with missing comorbidity metadata were excluded from this analysis. The described statistical comparison of vaccination schemes within the mix and match cohort was performed after the exclusion of recovered individuals.

To assess the impact of a previous SARS-CoV-2 infection on RBD antibody titres and wild-type ACE2 binding inhibition among the different vaccination schemes, we also used a two-sided generalized Wilcoxon (Brunner-Munzel) test.

To generate a heat map for comparing antigen-specific antibody formation across different vaccination schemes within the mix and match sample cohort, normalised antibody responses were initially scaled using the function “z-score”, before being plotted as a heat map. To evaluate longitudinal changes in antibody response and ACE2 binding inhibition within our longitudinal sample cohort, changes from T1 to T2 were calculated using log2 fold change. Any increase in titre or binding is represented by a positive value, while decreases in titre or binding are represented by negative values.

Data visualization was done in RStudio (Version 1.2.5001 running R version 3.6.1). Additional packages “gplots” (37) and “beeswarm” (38) were used for specific displays (22). Graphs were exported from RStudio and further edited in Inkscape (Version 0.92.4) to generate final figures.

## Results

**Figure 1:**
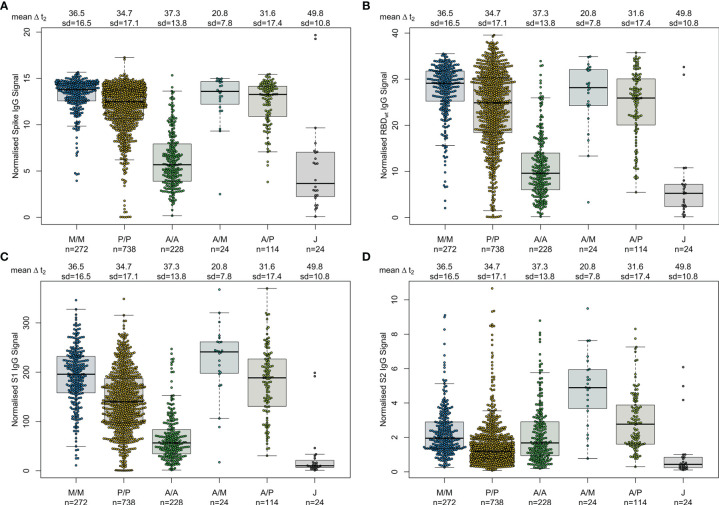
Different SARS-CoV-2 vaccination schemes result in distinct humoral responses. IgG antibody titres against full-length Spike trimer **(A)**, receptor-binding domain (RBD) **(B)**, S1 domain **(C)** and S2 domain **(D)** were measured with MULTICOV-AB. Individuals received either homologous mRNA-1273 (M/M, blue, n = 272), BNT162b2 (P/P, orange, n = 738) or AZD1222 (A/A, green, n = 228), heterologous AZD1222-mRNA-1273 (A/M, light blue, n = 24), AZD1222-BNT162b2 (A/P, light green, n = 114), or a single dose of Ad26.CoV2.S (J, grey, n = 24). Raw MFI values were normalised against QC samples to generate signal ratios for each antigen. Data is shown as box and whisker plots overlaid with strip charts. Boxes represent medians, 25th and 75th percentiles and whiskers show the largest and smallest non-outlier values based on 1.5 IQR calculation. Time between sampling and full vaccination is displayed as mean and SD for each group. Number of samples per vaccination scheme are stated below.

First, we examined differences in humoral responses between individuals who received homologous or heterologous immunisation schemes within our mix and match sample cohort where vaccine dose distribution is similar to the German vaccine coverage. Using MULTICOV-AB, we compared vaccination-induced antibody titres generated against the full-length Spike trimer, RBD, S1 and S2 domains and found that mRNA-based homologous vaccinations induced a greater Spike (median normalised MFI: mRNA-1273 13.78, BNT162b2 12.49, AZD1222 5.68, Ad26.CoV2.S 3.65), RBD (median normalised MFI: mRNA-1273 29.12, BNT162b2 24.89, AZD1222 9.61, Ad26.CoV2.S 5.25) and S1 response (median normalised MFI: mRNA-1273 195.9, BNT162b2 139.8, AZD1222 56.40, Ad26.CoV2.S 10.14) than vector-based ones (**Figure 1**). When comparing between the two vector-based vaccinations, the two-dose immunisation with AZD1222 resulted in higher titres than the one-dose Ad26.CoV2.S from Janssen. For mRNA vaccines, Moderna’s mRNA-1273 produced a significantly higher response than Pfizer’s BNT162b2 (p-values <0.001, **Supplementary Table 5**). Heterologous dose vaccination schemes resulted in comparable titres (for Spike and RBD) as homologous mRNA vaccine regimens among our study group independent of the origin of the second dose (Spike normalised MFI: AZD1222-mRNA-1273 13.59, AZD1222-BNT162b2 13.27, RBD normalised MFI: AZD1222-mRNA-1273 28.17, AZD1222-BNT162b2 25.93). Heterologous titres were in addition significantly higher than those after a homologous AZD1222 two-dose immunisation (p-values <0.001, **Supplementary Table 5**). In line with their lower titres, serological non-responder rate (defined as a Signal to Cutoff ratio (S/CO) below 1 for either Spike or RBD antigen) was highest for vector-based homologous vaccination schemes (**Table 2**).

**Table 2:** Vaccine non-responder rates across study population.

Sample cohort (n)	ΔT range post-vaccination (days)	Vaccine (n)	Non-responders MULTICOV-AB (n, %)	Non-responders ACE2-RBD WT (n, %)
**Mix and match (1400)**	7-65	M/M (272)	0 (0.0)	0 (0.0)
		P/P (738)	9 (1.2)	18 (2.4)
		A/A (228)	4 (1.8)	26 (11.4)
		A/M (24)	0 (0.0)	0 (0.0)
		A/P (114)	0 (0.0)	0 (0.0)
		J (24)	3 (12.5)	22 (91.7)
**Time points (597)**	5-12	P/P (107)	6 (5.6)	13 (12.2)
		M/M (40)	1 (2.5)	1 (2.5)
	26-30	P/P (103)	1 (1.0)	2 (1.9)
		M/M (8)	0 (0.0)	0 (0.0)
	54-58	P/P (92)	1 (1.1)	3 (3.3)
		M/M (22)	0 (0.0)	0 (0.0)
	94-103	P/P (139)	2 (1.4)	8 (5.8)
		M/M (7)	0 (0.0)	0 (0.0)
	129-146	P/P (38)	3 (7.9)	2 (5.3)
		M/M (5)	1 (20.0)	1 (20.0)
	176-203	P/P (36)	0 (0.0)	8 (22.2)
		M/M (0)	n. a.	n. a.
**Longitudinal (180)**	T1: 7-63	P/P T1 (90)	2 (2.2)	3 (3.3)
	T2: 121-203	P/P T2 (90)	2 (2.2)	17 (18.9)

MULTICOV-AB non-responders were determined as in (28), with samples that had a signal to cutoff ratio below 1 for either the Spike or RBD being considered non-responders. ACE2-RBD non-responders were determined as in (29), with samples that had a ACE2 binding inhibition less than 20% being considered non-responders. Different vaccines and combinations are abbreviated as follows: M/M (two-dose mRNA-1273), P/P (two-dose BNT162b2), A/A (two-dose AZD1222), A/M (first dose AZD1222, second dose mRNA-1273), A/P (first dose AZD1222, second dose BNT162b2) and J (one-dose Ad26.CoV2.S). The time points sample cohort contains only homologous BNT162b2 and mRNA-1273 samples. The longitudinal sample cohort contains only paired homologous BNT162b2 taken at time 1 (T1) or 2 (T2).

**Figure 2:**
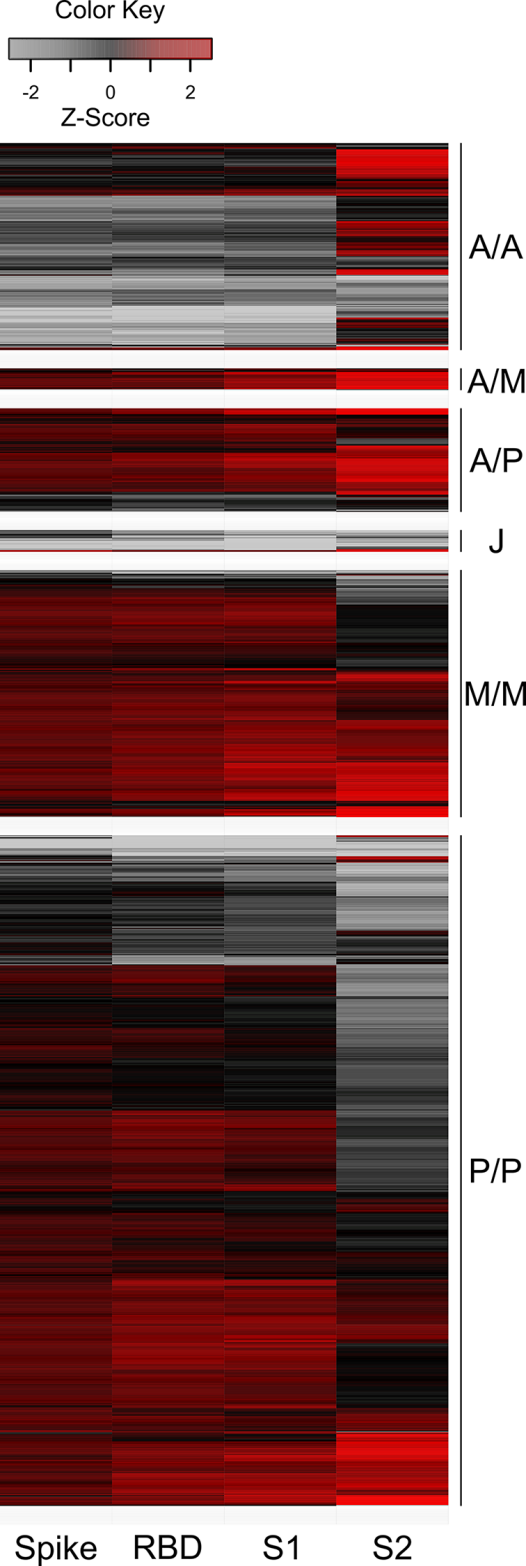
Humoral immune response after mRNA vaccination is skewed towards increased RBD and S1 titres, while vector-based vaccination results in increased S2 antibody levels. Antigen-specific antibody titres measured in the mix and match sample cohort were scaled and centered per antigen. Resulting values greater than 2.5 and smaller than -2.5 were set to these extreme values instead. Samples were then clustered within their subgroups based on immunisation scheme and are displayed as a heat map. Negative values represent below average titres and positive values represent positive above average titres per antigen. Colour shades indicate low (grey) to high (red) value distribution. A/A – two-dose AZD1222. A/M – first dose AZD1222, second dose mRNA-1273. A/P – first dose AZD1222, second dose BNT162b2. M/M – two-dose mRNA-1273. P/P – two-dose BNT162b2. J – one-dose Ad26.CoV2.S.

As multiplex-based serology tests such as MULTICOV-AB offer the unique opportunity for in-depth profiling of polyclonal antibody reactivity towards multiple viral antigens, we then assessed differences in antibody specificities between the different vaccines. Within the mix and match sample cohort, we observed that mRNA-based SARS-CoV-2 vaccinations resulted in reduced S2-specific antibody titres compared to vector-based ones (**Figure 1**). To investigate this unequal antibody distribution further, we initially scaled titres for each individual antigen (**Figure 2**), and found that while Spike, RBD, S1 titres were low for both AZD1222 and Ad26.CoV2.S, S2-specific titres were considerably higher than expected. We then calculated proportional ratios between antigens (**Table 3**), confirming that homologous mRNA vaccination resulted in significantly higher proportion of RBD- (mRNA-1273 14.01-fold, BNT162b2 18.63-fold, AZD1222 5.23-fold) and S1-targeted antibodies (mRNA-1273 97.21-fold, BNT162b2 110.10-fold, AZD1222 33.48-fold) compared to S2-targeted immunoglobulins. This over-representation of S1-targeting antibodies following mRNA vaccination, was also present in those who received a heterologous immunisation scheme (AZD1222-mRNA-1273 47.60-fold, AZD1222-BNT162b2 65.06-fold).

**Table 3:** Antigen-specific ratios for different vaccination schemes.

	Antibody target (95% CI)					
Vaccine	RBD vs S	S1 vs S	S1 vs RBD	S vs S2	RBD vs S2	S1 vs S2
A/A	1.72 (1.66-1.78)	10.09 (9.72-10.47)	5.89 (5.73-6.11)	3.38 (3.04-3.60)	5.23 (4.65-6.07)	33.48 (27.92-37.56)
A/M	1.97 (1.85-2.26)	16.71 (15.04-18.37)	8.15 (7.86-8.68)	2.76 (2.15-3.48)	5.86 (4.58-6.28)	47.60 (41.28-53.66)
A/P	1.97 (1.90-2.04)	14.16 (13.42-14.64)	7.17 (6.95-7.36)	4.76 (4.08-5.46)	8.60 (7.85-10.53)	65.06 (58.95-69.61)
M/M	2.10 (2.06-2.12)	14.19 (13.67-14.47)	6.80 (6.61-6.93)	6.88 (6.24-7.55)	14.01 (12.74-15.09)	97.21 (92.07-100.90)
P/P	2.00 (1.96-2.04)	11.21 (10.95-11.52)	5.72 (5.64-5.78)	9.99 (9.48-10.43)	18.63 (17.82-20.00)	110.10 (104.20-114.10)
J	1.23 (0.97-1.61)	3.30 (2.72-4.18)	2.79 (2.35-4.06)	8.89 (3.71-13.27)	6.83 (4.70-22.35)	31.53 (15.53-38.63)

Ratios were calculated by dividing normalised MFI values for the two targets for all samples. RBD – receptor-binding domain, S – full-length trimeric Spike protein. Median values with 95% CI in brackets are shown. A/A – two-dose AZD1222. A/M – first dose AZD1222, second dose mRNA-1273. A/P – first dose AZD1222, second dose BNT162b2. M/M – two-dose mRNA-1273. P/P – two-dose BNT162b2. J – one-dose Ad26.CoV2.S.

**Figure 3:**
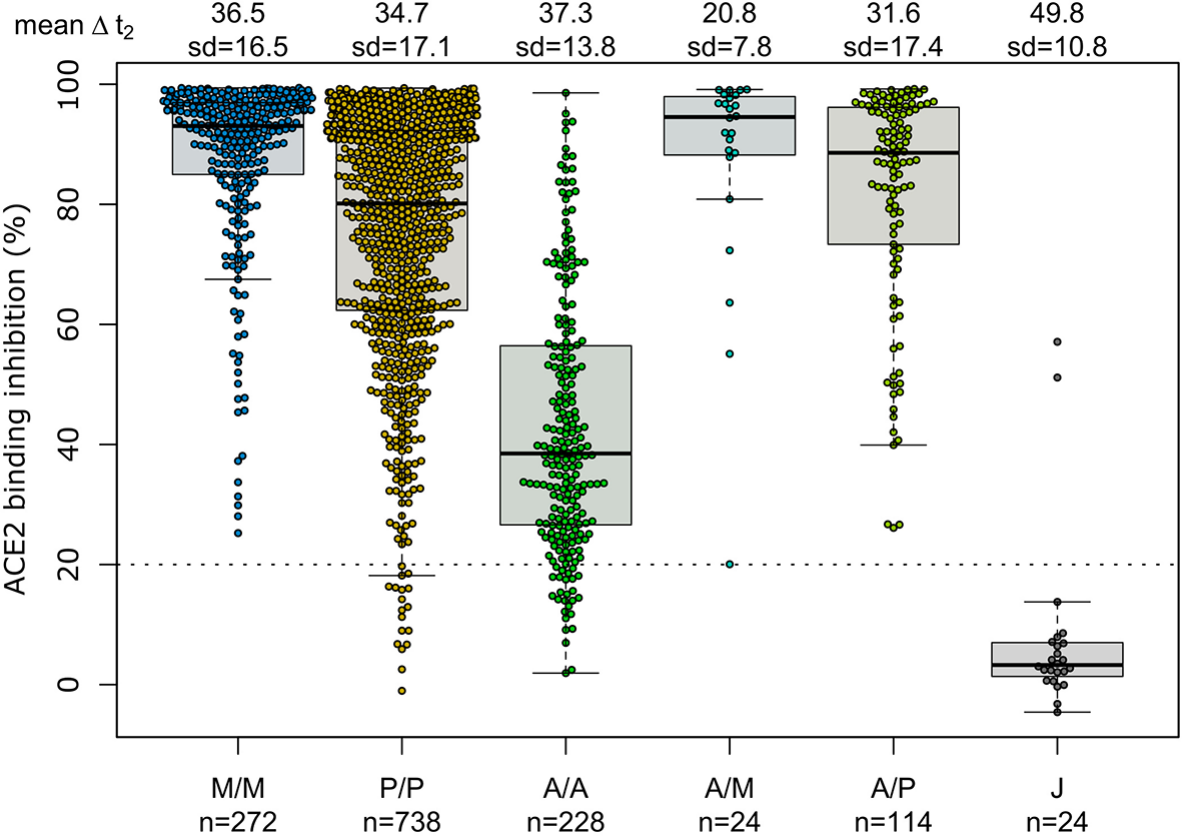
Different vaccination schemes impact ACE2 binding inhibition against SARS-CoV-2 wild-type. ACE2 binding inhibition against the SARS-CoV-2 wild-type (B.1 isolate) RBD was assessed by an ACE2-RBD competition assay for homologous mRNA [mRNA-1273 (M/M, blue), BNT162b2 (P/P, orange)], heterologous prime-boost (AZD1222-mRNA-1273 (A/M, light blue), AZD1222-BNT162b2 (A/P, light green) or vector-based [AZD1222-AZD1222 (A/A, green), Ad26.CoV2.S (J, grey)] vaccination schemes in the mix and match cohort. Data is shown as box and whisker plots overlaid with strip charts. Boxes represent medians, 25th and 75th percentiles and whiskers show the largest and smallest non-outlier values based on 1.5 IQR calculation. The threshold for non-responsive samples (ACE2 binding inhibition less than 20%) is shown as dotted line. All samples below this threshold can be considered non-responsive. Time between sampling and full vaccination is displayed as mean and SD for each group. Number of samples per vaccination scheme are stated below. ACE2 binding inhibition towards VoCs can be found in **Supplementary Figure 2**.

Having determined that mRNA vaccines produce a significantly higher proportion of RBD and S1 antibodies, we next investigated their ACE2 binding inhibition as these antigens are predominantly responsible for antibody-mediated virus neutralization (12, 13). For this, we used a previously published ACE2-RBD competition assay (22, 29, 30), which detects neutralizing antibody activity only and is comparable to classical viral neutralization assays (24, 29). As expected, homologous mRNA vaccination resulted in higher ACE2 binding inhibition than homologous vector-based vaccination (median ACE2 binding inhibition mRNA-1273 93.1%, BNT162b2 80.1%, AZD1222 38.5%, Ad26.CoV2.S 3.3%, **Figure 3**). Neutralizing antibodies generated following vaccination with Ad26.CoV2.S resulted in minimal ACE2 binding inhibition, with only 8.3% being classified as responders (29). As variants of concern now comprise the majority of infections globally (39), we also assessed ACE2 binding inhibition against the Alpha, Beta, Gamma and Delta SARS-CoV-2 VoC strains. ACE2 binding inhibition was most similar to wild-type for the Alpha variant, followed by Delta whereas Beta and Gamma variants had the largest reductions in ACE2 binding inhibition (**Supplementary Figure 2**).

**Figure 4:**
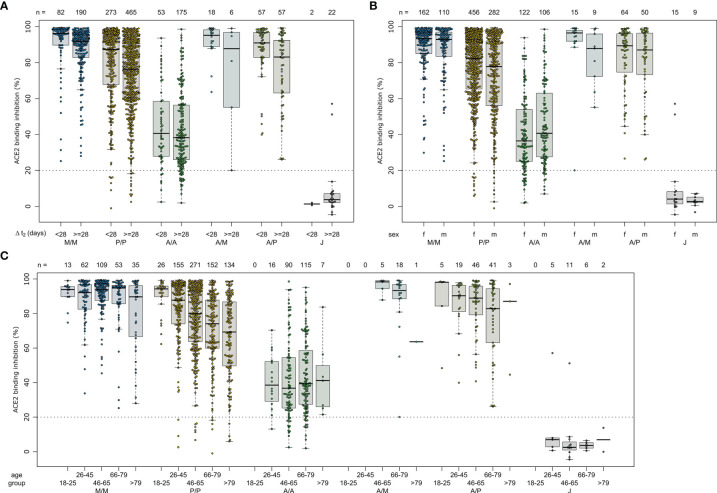
Effect of time post-vaccination, gender and age on ACE2 binding inhibition for different SARS-CoV-2 vaccination schemes. ACE2 binding inhibition against the SARS-CoV-2 wild-type (B.1 isolate) RBD was assessed by an ACE2-RBD competition assay. Samples were split according to vaccination scheme (homologous mRNA [mRNA-1273 (M/M, blue), BNT162b2 (P/P, orange)], heterologous prime-boost (AZD1222-mRNA-1273 (A/M, light blue), AZD1222-BNT162b2 (A/P, light green) or vector-based [AZD1222-AZD1222 (A/A, green), Ad26.CoV2.S (J, grey)]. To display impact of potential confounders, samples were further split in time post-vaccination up to 27 and above 27 days **(A)**, gender **(B)** and indicated age groups **(C)**. Boxes represent medians, 25th and 75th percentiles and whiskers show the largest and smallest non-outlier values based on 1.5 IQR calculation. The threshold for non-responsive samples (ACE2 binding inhibition less than 20%) is shown as dotted line. All samples below this threshold can be considered non-responsive. Time between sampling and full vaccination is displayed as mean and SD for each group. Number of samples per vaccination scheme are stated below the figure. Statistical significance was calculated by a regression model (**Supplementary Table 4**).

Due to the range of responses recorded for each dose combination and likely differences in population characteristics as a result of changing vaccine recommendations, we examined whether confounders (sampling time post-vaccination (ΔT), age, gender or comorbidities) were instead responsible. To analyze impact of ΔT, we separated samples into 7 to 27 days post-final dose to capture peak response and 28 to 65 days post-final dose to capture plateau response (**Figure 4**). While there was a reduction in median response for samples from individuals collected within the plateau phase, the pattern between the vaccines remained consistent. While increasing age did result in small reductions in ACE2 binding inhibition (only significant for BNT162b2, p<0.001), the vaccine dosing scheme received had a substantially larger effect, with the eldest age group (>79) of homologous mRNA vaccine recipients still having increased IgG titres and ACE2 inhibition capacities than the youngest (26 to 45) AZD1222 recipients (**Figure 4**). Regression modelling for ACE2 binding inhibition against wild-type confirmed the decrease of ACE2 binding inhibition with time post-vaccination for all vaccination types except homologous AZD1222 (**Supplementary Table 4A**). While age did not cause a significant decrease for homologous AZD1222, this may have been due to the low number of samples at both ends of the age range within our cohort. For mRNA-1273, while age did result in a significant decrease during the peak period (p=0.029), this was not present within the plateau phase (p=0.615). For homologous BNT162b2 vaccination, male sex seemed to be associated with a decreased ACE2 binding inhibition, although the same was not true for mRNA-1273. Similar patterns were observed for the ACE2 binding inhibition against Alpha, Beta, Gamma and Delta VoCs (**Supplementary Tables 4B–E**). As we observed serological non-responders within our mix and match study cohort, we systematically evaluated their distribution among the different immunisation schemes (**Table 2**). Overall, vector-based homologous vaccination (2.8%) resulted in a higher proportion of non-responders than homologous mRNA-based vaccination (0.9%). Neither age nor gender was a determining factor in being a non-responder.

**Figure 5:**
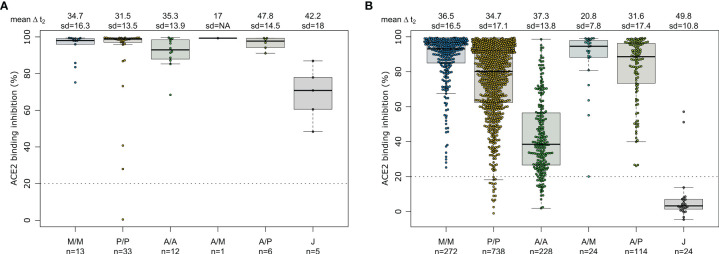
SARS-CoV-2 vaccination boosts ACE2 binding inhibition among recovered individuals independent of vaccination scheme. Differences in vaccination responses of recovered previously SARS-CoV-2 infected individuals from our mix and match cohort were analyzed using an ACE2-RBD competition assay **(A)**. SARS-CoV-2 infection status was based on a previous self-reported positive PCR/antigen test or a MULTICOV-AB nucleocapsid IgG normalisation ratio above 1. Samples were split according to vaccination scheme in homologous mRNA (mRNA-1273 (M/M, blue), BNT162b2 (P/P, orange)), heterologous prime-boost (AZD1222-mRNA-1273 (A/M, light blue), AZD1222-BNT162b2 (A/P, light green) or vector-based (AZD1222-AZD1222 (A/A, green), Ad26.CoV2.S (J, grey)). For clarity and comparison, ACE2 inhibition of SARS-CoV-2 naïve individuals are displayed **(B)**. Boxes represent medians, 25th and 75th percentiles and whiskers show the largest and smallest non-outlier values based on 1.5 IQR calculation. If not enough sample to generate a box were present (minimum 5), then only the median is indicated by a line. The threshold for non-responsive samples (ACE2 binding inhibition less than 20%) is shown as dotted line. All samples below this can be considered non-responsive. Time between sampling and full vaccination is displayed as mean and SD for each group. Number of samples per vaccination scheme are stated below the graph. Results of a formal statistical comparison of recovered-vaccinated and SARS-CoV-2 naïve-vaccinated individuals are shown in **Supplementary Table 6**.

As our population-based cohort also contained individuals who had been previously infected and then vaccinated, we examined what effect this had upon their vaccine-induced response. As previously observed (40), recovered and then vaccinated individuals developed high levels of IgG with strong ACE2 binding inhibition (**Figure 5** and **Supplementary Table 6**). While mRNA or heterologous vaccination of recovered individuals still elevated median RBD IgG titres (mRNA-1273 31.81, BNT162b2 32.23, AZD1222-mRNA-1273 35.18, AZD1222-BNT162b2 30.49, **Supplementary Figure 3**) and median ACE2 binding inhibition (mRNA-1273 98.1%, BNT162b2 98.8%, AZD1222-mRNA-1273 99.3%, AZD1222-BNT162b2 97.7%, **Figure 5**), increases were particularly apparent for the vector-based vaccinations where median RBD IgG titres (AZD1222 24.69, Ad26.CoV2.S 36.53, **Supplementary Figure 3**) and median ACE2 binding inhibition (AZD1222 92.9%, Ad26.CoV2.S 70.8%, **Figure 5**) were significantly higher than in SARS-CoV-2 naïve vaccinated individuals (median RBD IgG mRNA-1273 29.12, BNT162b2 24.89, AZD1222-mRNA-1273 28.17, AZD1222-BNT162b2 25.93, AZD1222 9.61, Ad26.CoV2.S 5.25, **Figure 1**) and median ACE2 binding inhibition (mRNA-1273 93.1%, BNT162b2 80.1%, AZD1222-mRNA-1273 94.5%, AZD1222-BNT162b2 88.6%, AZD1222 38.5%, Ad26.CoV2.S 3.3%, **Figure 3**).

**Figure 6:**
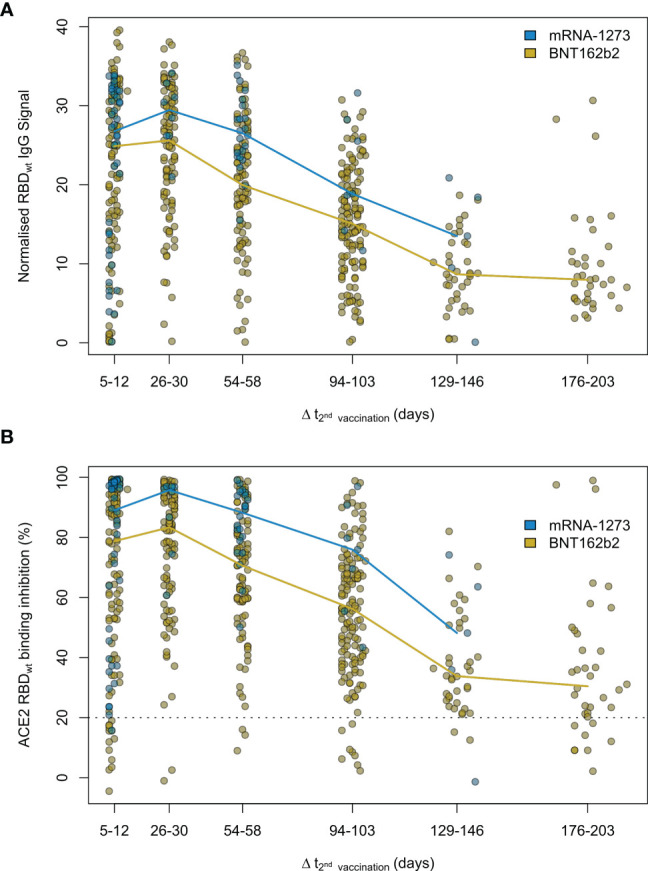
Antibody and neutralization response kinetic up to 7 months after SARS-CoV-2 mRNA vaccination. Humoral vaccine response was assessed by MULTICOV-AB **(A)** and an ACE2-RBD competition assay **(B)** using the time point sample set. Samples were either 5 to 12, 26 to 30, 54 to 58, 94 to 103, 129 to 146 and 176 to 203 days post-second dose of either a two-dose BNT162b2 (yellow, n = 515) or mRNA-1273 (blue, n = 82) vaccination. Colored line connects median response per time point and vaccine. Data is displayed as normalised IgG RBD ratio or as % ACE2 binding inhibition where 100% indicates maximum binding inhibition and 0% no binding inhibition. The threshold for non-responsive samples (ACE2 binding inhibition less than 20%) is shown as dotted line. All samples below this threshold can be considered non-responsive. Median values with 95% CI and IQR of RBD_wt_ IgG signal and ACE2 binding inhibition are shown in **Supplementary Table 7**.

**Figure 7:**
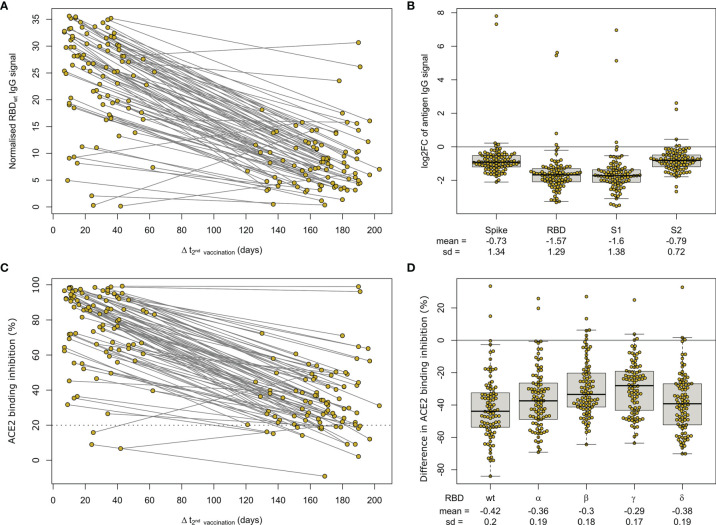
Longitudinal mRNA vaccine response monitoring defines decreases over time. Humoral response was assessed by MULTICOV-AB **(A, B)** and an ACE2-RBD competition assay **(C, D)** for all samples of the paired longitudinal sample set at indicated time points post-second dose of 90 individuals after a two-dose BNT162b2 vaccination. Line graph illustrates longitudinal development of RBD-specific antibody levels **(A)** or ACE2 binding inhibition **(C)** towards WT RBD as time post-vaccination increases. The threshold (less than 20% ACE2 binding inhibition) for non-responsive samples within the ACE2-RBD competition assay is shown (dotted line). All samples below this threshold are considered non-responsive. SARS-CoV-2 antigen-specific differences in longitudinal humoral response are expressed as log2 fold changes **(B)**. Reduction in ACE2 binding inhibition for RBD of Alpha, Beta, Gamma and Delta VoC between sampling times is shown as difference **(D)**.

Having determined that mRNA-based vaccination resulted in an increased humoral response, we evaluated lifespan and antibody response kinetics using our time point sample cohort which were selected to mimic key response periods for antibody-producing B-cell activity such as expansion, peak and plateau phase after a complete vaccination scheme. Vaccine-induced titres and ACE2 binding inhibition both initially increased, peaked during the second time point (26 to 30 days post-second dose), and then decreased linearly as time increased (**Figure 6** and **Supplementary Table 7**). ACE2 binding inhibition followed the same pattern of decrease as time increased. In contrast to antibody levels, the percentage of non-responders showed however a trend for increased decline already from time point 94 to 103 days post-second vaccination onwards for BNT162b2, with 22.2% of samples considered as non-responders at 176-203 days post-second vaccination (**Table 2**). As already observed in **Figure 1**, mRNA-1273 (blue line) resulted in higher titres and ACE2 binding inhibition compared to BNT162b2 (yellow line) for all monitored time points. To validate this pattern of decreasing antibody titres and ACE2 inhibition activity, we examined samples from a cohort of longitudinal donors (longitudinal sample cohort). Unlike the time point sample cohort, this cohort contained paired samples from each donor which allows to directly compare changes in titre and neutralization activity from the first sampling to the second sampling. While these samples had a variable initial ΔT post-full vaccination (7-63 days), the sampling intervals between first and second donation were more comparable (114-163 days). Overall, mean reduction in RBD-specific antibody titres were 66.3% between their first and second sampling (**Figure 7**). Among the different SARS-CoV-2 antigens, RBD and S1 antibodies had the largest decrease, while Spike Trimer and S2 had the smallest. This reduction in titre was also reflected in ACE2 binding inhibition from the first to second sampling for both the wild-type RBD which was reduced substantially (mean difference of 42%) and for all the VoC RBDs (mean difference: Alpha 36%, Beta 30%, Gamma 29%, Delta 38%).

## Discussion

We report both significant and substantial differences in humoral responses generated by the different vaccines and immunisation schemes currently available in Germany, with homologous mRNA or combined heterologous vector and mRNA vaccination approaches inducing significantly higher titres and ACE2 binding inhibition compared to homologous vector-based vaccination schemes. This expands on results from on-going randomized and observational trials such as the ComCoV (41) or CoCo (42) study which provided only information on AZD1222-BNT162b2 schemes (20, 43). Further, as expected titres and ACE2 binding inhibition for AZD1222 were reduced compared to mRNA-based vaccination (43). Among homologous mRNA regimens, we identified like others, that mRNA-1273 resulted in higher antibody titres and ACE2 binding inhibition than BNT162b2 (40, 44). Extending time periods between successive doses of mRNA and vector-based vaccinations also positively impacted on serological and cellular response levels or vaccine efficiency and effectiveness (1, 45–49). The German STIKO recommended at the time of the study dosing intervals of six weeks between mRNA vaccines, 12 weeks for vector vaccines and 9-12 weeks for heterologous vaccination approaches (18). While heterologous and vector vaccination dosing intervals in our mix and match cohort adhere more closely to those extended intervals (1), time periods for mRNA vaccine dosing across our study population mimic 21 or 28 days from clinical trials (3, 4) and licensing agreements (50, 51) making them unlikely contributors to the observed differences in humoral responses. While we have used an ACE2-RBD competition assay to measure ACE2 binding inhibition as opposed to classic virus neutralization assays, this assay analyses neutralizing antibodies as seen by its similar performance to VNT (24, 29). ACE2 inhibition assays instead of a VNT have also already been used successfully by other groups to determine neutralizing antibody activity (52). Methodically, MULTICOV-AB and the ACE2-RBD competition assay are also complementary and are measured using a single initial sample dilution which further reduces variability between their results. By multiplex-based antibody profiling, we were able to further investigate titre differences and determined that vector- and mRNA-based vaccines induced a distinct pattern of Spike subdomain-targeted antibodies. While vector-based formulations result in a significantly larger proportion of S2-domain antibodies, RBD- and S1-domain antibodies dominated in mRNA vaccines. While these observations require further detailed investigation, the relative over-representation of RBD- and S1-targeting antibodies within mRNA vaccines is particularly intriguing as these two antigens comprise the majority of neutralizing antibody activity (13, 53). Although a series of modelling studies have now linked levels of neutralizing antibodies to vaccine efficacy (12, 54), a clearly defined correlation of vaccine efficacy and neutralizing antibody levels is still lacking. Nevertheless it appears logical that increased antibody levels specific to virus proteins-mediating cell attachment could result in enhanced levels of protection from infection and contribute to observed differences in vaccine efficacy and effectiveness levels (3, 55, 56). Interestingly, our conclusions are strengthened by studies examining the relative immunogenicity of the different Spike subdomains. By immunising rabbits with SARS-CoV-2 S2, S1 or RBD proteins, Ravichandran et al. were able to show that S2 protein elicited considerable lower neutralizing antibody levels compared to the S1 and RBD antigens (57). Similar results were obtained from isolating immunoglobulins of COVID-19 convalescents where S2 subunit-targeting antibodies showed weaker SARS-CoV-2 neutralization activity compared to the RBD-targeting ones (58). While the Spike protein surface is extensively glycosylated, including the membrane-proximal S2 domain, the RBD completely lacks N-glycans which might explain its immunodominance (59–61).

An additional finding of our study requiring further investigation is the relatively poor performance of Ad26.CoV2.S, particularly for induction of neutralizing antibodies for both SARS-CoV-2 wild-type and VoC RBDs. While some studies have reported sufficient levels of neutralizing activity after vaccination with Ad26.CoV2.S (2), others identified minimal neutralizing activity, particularly when compared to other COVID-19 vaccines from Pfizer or Moderna (44). The relatively poor performance of Ad26.CoV2.S in inducing an antibody response has also been identified by researchers studying other bodily fluids (e.g. breast milk), who found that Ad26.CoV2.S produced significantly fewer IgA antibodies than BNT162b2 or mRNA-1273 (62). While our Ad26.CoV2.S sample group size is low (n=29), it is three times larger than a recent study from the manufacturer which reported neutralizing activity against Delta and other VoCs [n=8 (63)]. It should be noted that four of the eight individuals within their cohort were reported as being spike seropositive at baseline which is a consistent finding with our cohort, where strong ACE2 binding inhibition was only achieved in those individuals who had been previously infected. Our median time point is however earlier than the reported peak of antibody activity (2, 64). Further independent investigations into the neutralizing activity generated by single-dose Ad26.CoV2.S to clarify those differing results within SARS-CoV-2 naïve individuals are therefore urgently needed.

Among confounding variables, we identified like others that age resulted in a general reduction in titre and ACE2 binding inhibition (11, 40, 65), although the vaccination scheme received had a more significant effect. While recovered individuals developing high titres and ACE2 binding inhibition once vaccinated has been previously reported (40, 43), we found that these responses were similar among all vaccines and immunisation schemes. Given that current German guidelines require a six month post-positive PCR waiting period before receiving a first dose, this suggests that such individuals would be suitable for all currently licensed vaccines, assuming they meet pre-existing EMA and STIKO criteria. This ability to use all vaccines and generate a substantial response will be of particular public health importance, given the on-going booster dose administration which could impact availability for some vaccine brands, as happened earlier in 2021.

Our results on the longevity of the humoral response post-vaccination is similar to others, in identifying an initial peak from approximately 28 days post-second dose onwards followed by a gradual reduction over time (66). As expected, ACE2 binding inhibition and titre are mostly mirrored in their decline over time. However, the increased numbers of non-responders from BNT162b2-vaccinated individuals from six months after the second vaccine needs further careful monitoring until a precise correlate of protection has been defined. Among the different SARS-CoV-2 antibodies, it is unsurprising that the RBD and S1 underwent the greatest reductions as they had the largest titres to begin with. Between VoCs, we did not identify any apparent differences in ACE2 binding inhibition between the differing immunisation schemes for confounders. Instead, again vaccine type or regimen (homologous vs heterologous) received had the largest effect upon ACE2 binding inhibition. The VoCs themselves followed a previously published pattern (9, 22, 67), with the lowest reduction for the Alpha variant, and the highest for the Beta and Gamma variants. It should be stated that in our analysis of longitudinal samples, there is a wide variety of timeframes post-vaccination, meaning that initial samples are collected both before, during and after the initial peak response at around 28 days. While we have then made the assumption that decreases in responses would be linear to the second sampling, this is not the case as some of the early collected samples (e.g. 7 days post-second vaccination) would have initially increased before later decreasing. However, our purpose of this analysis was to measure changes over a larger timeframe (4 months) and the difference in time from first to second sampling, means that all samples should be in the decline phase by their second sampling.

Our manuscript has several limitations, namely that we are only measuring antibodies (including neutralizing antibodies) that are present within serum. As previously stated, we have used an ACE2-RBD competition assay to measure inhibition of ACE2 binding instead of classical virus neutralization assays, although the results of this assay have already been shown to be similar to VNT and are known to be specific to neutralizing antibody responses only. While neutralizing antibodies themselves are considered a strong correlate for protection (13), other components that are not measured within our assays such as T-cell mediated immunity will also offer protection (68, 69). Our use of serum also means that memory B-cells, which are involved in protection against severe disease progression (70), are equally excluded from our analysis. Our study cohort consists of relatively low sample numbers for both heterologous and Ad26.CoV2.S vaccinations whereas BNT162b2 samples are overrepresented. However, our sample numbers are similar or in case of Ad26.CoV2.S exceed other previously published work making our study one of the largest independent evaluation studies of this vaccine. Our BNT162b2 sample size mimics dose distribution in Germany where approximately 70% of delivered vaccine doses were from Pfizer. Our study population is also relatively similar in regard to age and gender. Last, self-reported information about a previous SARS-CoV-2 infection or vaccination could bias study outcome. However, recent studies have found a good correlation between self-reported and administrative records with 98% consistency for vaccination type and 95% for vaccination date or detection of SARS-CoV-2 antibodies with a positive predictive value of 98.2% and a negative predictive value of 97.3%, respectively (71, 72). Additionally, our data on persistence and magnitude of vaccine-induced humoral responses is consistent with several other cohort-based studies (11, 20, 41, 66) which did not rely on self-reported vaccination records, therefore stressing the validity of our approach.

Next to an increasing number of observational studies including ours which examine vaccine-induced protection by assessing levels of humoral immunity, several large scale test-negative design (TND) studies have by now been conducted to determine vaccine effectiveness against a laboratory-confirmed SARS-CoV-2 infection requiring medical attention outside of randomized clinical trials (45, 73, 74). While readouts between those study types are fundamentally different and both are subject to different limitations (75–79), results are comparable. For instance, our findings of significantly higher titres and ACE2 binding inhibition after mRNA or heterologous immunisation schemes compared vector-based ones also translate to differing levels of vaccine effectiveness against SARS-CoV-2 infection of above 90% with at least one mRNA vaccine dose or of less than 70% with two doses of AZD1222 in a TND study from Canada (45). Equally, our results of decreasing humoral response levels after a full BNT162b2 vaccination correlate with increases in PCR-confirmed SARS-CoV-2 infections up to six month post-vaccination with BNT162b2 in an Israeli TND study (73).

Overall, we provide data on the vaccine-induced humoral response for all currently available mRNA-, vector-based and heterologous immunisation schemes in Germany. Within our population-based cohort, mRNA homologous or heterologous vaccination resulted in increased humoral responses. Our multiplex approach identified differences in quantities and ratios of RBD- and S1-targeting antibodies following mRNA homologous or heterologous vaccination. Further investigation into this targeting will be of particular interest to improve vaccine performance particularly for next generation vector-based vaccines.

## Data Availability Statement

Source data and the analysis code have been deposited on GitHub (https://github.com/BeckerMatthias/MuSPAD_VaccStudy).

## Ethics Statement

The studies involving human participants were reviewed and approved by the Ethics Committee of Hannover Medical School (9086_BO_S_2020). The participants provided their written informed consent to participate in this study.

## Author Contributions

MS, AD, BL, GK, and NS-M conceived the study. NS-M, MS, BL, VM, SC, and GK procured funding. AD, MS, MB, and NS-M designed the experiments. AD, JG, JJ, and DJ performed the experiments. AD, MS, MB, MH, JO, SC, NW, SG, J-KH, and BK performed data collection and analysis. AD, MB, MH, and BK generated figures and tables. TT, KF, TI, and NK organized sample collection and processing. AD, MS, MH, and BK verified the underlying data. AD, MS, and MB wrote the manuscript. AD, MH, JO, PH, BL, NS-M, MS, YK, DG, VM, DJ, PK, BT, UR, TT, KF, NK, TI, TK, AR, CS, AR, AM, and NG contributed resources or were involved in project administration. All authors critically reviewed and approved the final manuscript.

## Funding

This work was financially supported by the Initiative and Networking Fund of the Helmholtz Association of German Research Centres (grant number SO-96), the EU Horizon 2020 research and innovation program (grant agreement number 101003480 - CORESMA), intramural funds of the Helmholtz Centre for Infection Research and the State Ministry of Baden-Württemberg for Economic Affairs, Labour and Tourism (grant numbers FKZ 3-4332.62-NMI-67 and FKZ 3-4332.62-NMI-68). The funders had no role in study design, data collection and analysis, decision to publish, or preparation of the manuscript.

## Conflict of Interest

NS-M was a speaker at Luminex user meetings in the past. The Natural and Medical Sciences Institute at the University of Tübingen is involved in applied research projects as a fee for services with the Luminex Corporation.

The remaining authors declare that the research was conducted in the absence of any commercial or financial relationships that could be construed as a potential conflict of interest.

## Publisher’s Note

All claims expressed in this article are solely those of the authors and do not necessarily represent those of their affiliated organizations, or those of the publisher, the editors and the reviewers. Any product that may be evaluated in this article, or claim that may be made by its manufacturer, is not guaranteed or endorsed by the publisher.
