# Supplementary material for: Comparative Magnitude and Persistence of Humoral SARS-CoV-2 Vaccination Responses in the Adult Population in Germany
Source: Front Immunol. 2022 Feb 16;13:828053. doi: 10.3389/fimmu.2022.828053 (PMC8888837; doi:10.3389/fimmu.2022.828053)
Supplement: Supplementary file 1 [file DataSheet_1.zip › Supplementary Material -Comparative magnitude and persistence of humoral SARS-CoV-2 vaccination responses in the adult population in Germany.pdf]

Supplementary Material - Comparative magnitude and persistence of humoral SARS-CoV-2 vaccination responses in the adult population in Germany

Alex Dulovic[1,#], Barbora Kessel[2,#], Manuela Harries[2,#], Matthias Becker[1], Julia Ortmann[2], Johanna Griesbaum[1], Jennifer Jüngling[1], Daniel Junker[1], Pilar Hernandez[2], Daniela Gornyk[2], Stephan Glöckner[2], Vanessa Melhorn[2], Stefanie Castell[2], Jana-Kristin Heise[2], Yvonne Kemmling[2], Torsten Tonn[3], Kerstin Frank[3], Thomas Illig[4], Norman Klopp[4], Neha Warikoo[2], Angelika Rath[2], Christina Suckel[2], Anne Ulrike Marzian[2], Nicole Grupe[2], Philipp D. Kaiser[1], Bjoern Traenkle[1], Ulrich Rothbauer[1,5], Tobias Kerrinnes[6], Gérard Krause[2,7,8], Berit Lange[2,8,§,*], Nicole Schneiderhan-Marra[1,§,*], Monika Strengert [2,7,§,*]

[1] NMI Natural and Medical Sciences Institute at the University of Tübingen, Reutlingen, Germany
[2] Helmholtz Centre for Infection Research, Department of Epidemiology, Braunschweig, Germany
[3] German Red Cross Blood Donation Service North East, Dresden, Germany
[4] Hannover Unified Biobank, Hannover Medical School, Hannover, Germany
[5] Pharmaceutical Biotechnology, Department of Pharmacy and Biochemistry, University of Tübingen, Tübingen, Germany
[6] Helmholtz Institute for RNA-based Infection Research, Department of RNA-Biology of Bacterial Infections, Würzburg, Germany
[7] TWINCORE, Centre for Experimental and Clinical Infection Research, a joint venture of the Hannover Medical School and the Helmholtz Centre for Infection Research, Hannover, Germany
[8] German Centre for Infection Research (DZIF), partner site Hannover-Braunschweig, Braunschweig, Germany

# these authors have contributed equally to this work and share first authorship.
§ these authors have contributed equally to this work and share last authorship.
* corresponding authors.

* Correspondence:

Monika Strengert
monika.strengert@helmholtz-hzi.de

Berit Lange
berit.lange@helmholtz-hzi.de

Nicole Schneiderhan-Marra
nicole.schneiderhan@nmi.de

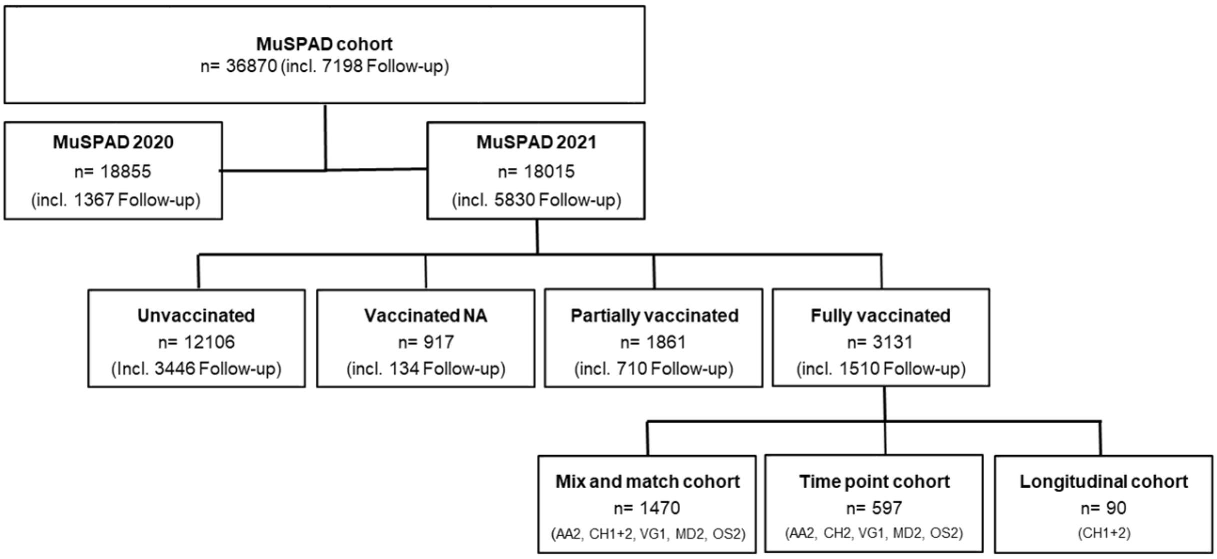

Supplementary Figure 1. Flow chart for sample selection from MuSPAD cohort. Sample selection for our vaccine response study from the entire MuSPAD cohort is displayed in a flow chart. Samples were selected based on selection criteria outlined in method section to examine impact of vaccination scheme, length and persistence of antibody response. Samples originated from the following study locations: Aachen 2 (AA2), Chemnitz 1(CH1), Chemnitz 2 (CH2), Vorpommern-Greifswald 1 (VG1), Osnabrück 2 (OS2), and Magdeburg 2 (MD2). Number of participants is given per group (n), NA: not available.

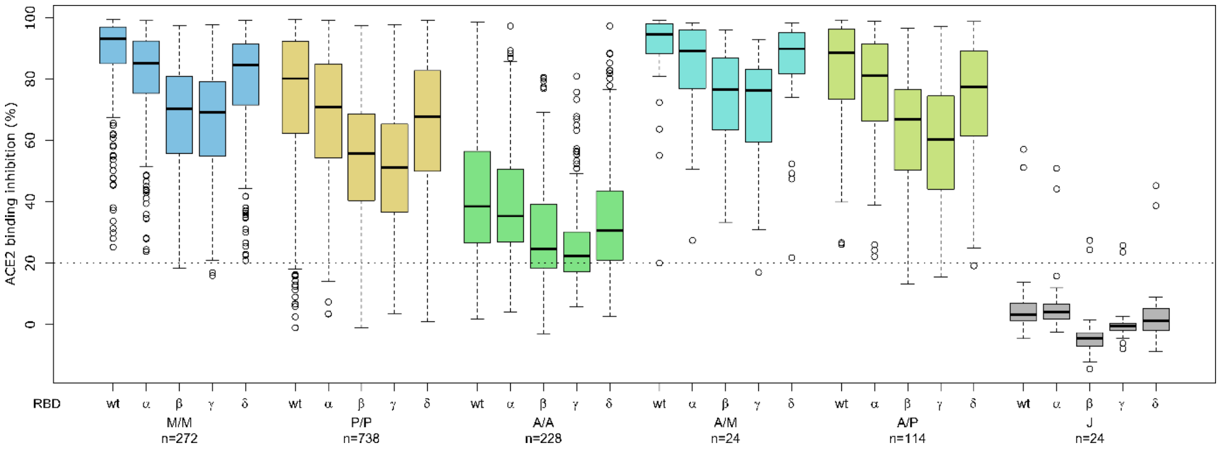

Supplementary Figure 2. Different VoCs reduce antibody ACE2 binding inhibition comparably between SARS-CoV-2 vaccination schemes.

ACE2 binding inhibition against the SARS-CoV-2 VoC Alpha (B.1.1.7), Beta (B.1.351), Gamma (P1) and Delta (B.1.617.2) RBDs was assessed by an ACE2-RBD competition assay for homologous mRNA (mRNA-1273 (M/M, blue), BNT162b2 (P/P, orange)), heterologous prime-boost (AZD1222-mRNA-1273 (A/M, light blue), AZD1222-BNT162b2 (A/P, light green) or vector-based (AZD1222-AZD1222 (A/A, green), Ad26.CoV2.S (J, grey)) vaccination schemes in the mix and match cohort. SARS-CoV-2 RBD WT (Figure 3) is again shown for clarity and comparison. Data is shown as box and whisker plots. Boxes represent medians, 25th and 75th percentiles and whiskers show the largest and smallest non-outlier values based on 1.5 IQR calculation. Outlier values are shown in clear circles. The threshold for non-responsive samples (ACE2 binding inhibition less than 20%) is shown as dotted line. All samples below this can be considered non-responsive. Number of samples per vaccination scheme are stated below the figure.

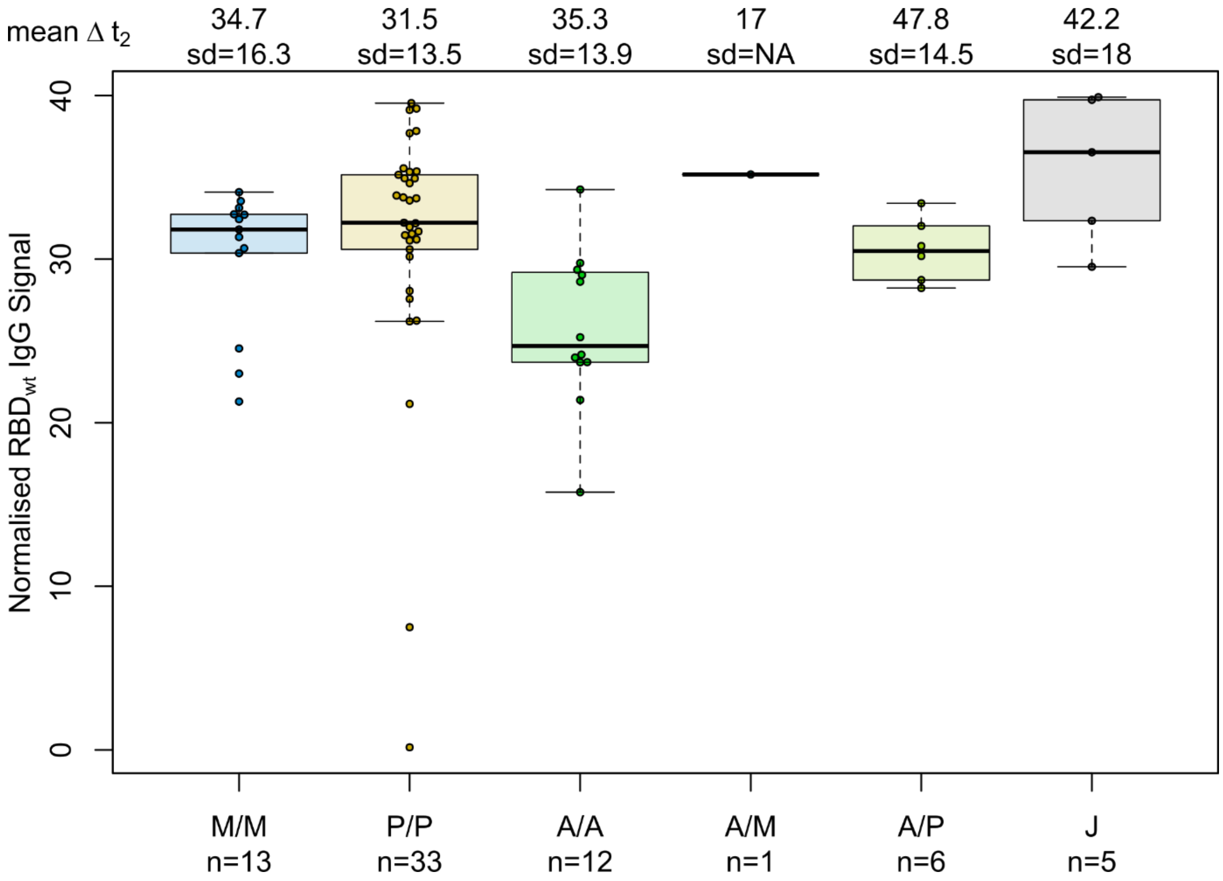

Supplementary Figure 3. SARS-CoV-2 vaccination boosts humoral response among recovered individuals independent of vaccination scheme.

Differences in vaccination responses of recovered previously SARS-CoV-2 infected individuals from our mix and match cohort were analyzed using MULTICOV-AB. SARS-CoV-2 infection status was based on a previous self-reported positive PCR/antigen test or a MULTICOV-AB nucleocapsid IgG normalisation ratio above 1. Samples were split according to vaccination scheme (homologous mRNA (mRNA-1273 (M/M, blue), BNT162b2 (P/P, orange)), heterologous prime-boost (AZD1222-mRNA-1273 (A/M, light blue), AZD1222-BNT162b2 (A/P, light green) or vector-based (AZD1222-AZD1222 (A/A, green), Ad26CoV2.S (J, grey)). Boxes represent medians, 25th and 75th percentiles and whiskers show the largest and smallest non-outlier values based on 1.5 IQR calculation. When less than 5 samples were available per group, only the median is indicated by a line. Time between sampling and full vaccination is displayed as mean and SD for each group. Number of samples per vaccination scheme are stated below the figure. Results of a formal statistical comparison of recovered-vaccinated and SARS-CoV-2 naïve-vaccinated individuals is shown in Table S6.

Supplementary Table 1. Comorbidities in study participants (n. a.: not applicable; NA: not available; CVD: cardiovascular disease).

Sample cohort (n)	SARS-CoV-2 infection status (n) *	ΔT range in days post-vaccination	Vaccine (n)	Hyper-tension (n, %)	CVD (n, %)	Diabetes (n, %)	Lung disease (n, %)	Cancer (n, %)	Immuno-suppression (n, %)
**Mix and match (1470)**	+ (70)	7-65	M/M (13)	4 (30.8)	1 (7.7)	2 (15.4)	0 (0.0)	0 (0.0)	0 (0.0)
			P/P (33)	12 (36.0)	4 (12.0)	3 (9.1)	0 (0.0)	0 (0.0)	1 (3.0)
			A/A (33)	7 (58.3)	3 (25.0)	2 (16.7)	1 (8.3)	1 (8.3)	2 (16.7)
			A/M (1)	1 (100.0)	0 (0.0)	0 (0.0)	0 (0.0)	0 (0.0)	0 (0.0)
			A/P (6)	3 (50.0)	1 (16.7)	0 (0.0)	1 (16.7)	0 (0.0)	0 (0.0)
			J (5)	2 (40.0)	0 (0.0)	0 (0.0)	0 (0.0)	0 (0.0)	1 (20.0)
	- (1400)		M/M (272)	84 (30.9)	23 (8.5)	13 (4.8)	17 (6.3)	2 (0.7)	11 (4.0)
			P/P (734, 4 NA)	263 (35.8)	101 (13.8)	64 (8.7)	59 (8.1)	20 (2.7)	37 (5.0)
			A/A (228)	91 (39.9)	25 (11.1)	25 (11.0)	12 (5.3)	2 (0.9)	9 (4.0)
			A/M (24)	12 (50.0)	4 (16.7)	2 (8.3)	0 (0.0)	1 (4.2)	0 (0.0)
			A/P (114)	43 (37.7)	16 (14.0)	10 (8.8)	10 (8.8)	4 (3.5)	6 (5.3)
			J (24)	9 (37.5)	2 (8.3)	2 (8.3)	2 (8.3)	1 (4.2)	2 (8.3)
**Time points (597)**	-	5-12	P/P (107)	50 (46.7)	16 (15.0)	6 (5.6)	3 (2.8)	0 (0.0)	7 (6.5)
			M/M (40)	13 (32.5)	3 (7.5)	3 (7.5)	1 (2.5)	0 (0.0)	1 (2.5)
		26-30	P/P (102; 1 NA)	31 (30.4)	15 (14.7)	11 (10.8)	12 (11.8)	0 (0.0)	0 (0.0)
			M/M (8)	3 (37.5)	1 (12.5)	0 (0.0)	0 (0.0)	0 (0.0)	0 (0.0)
		54-58	P/P (92)	37 (40.2)	14 (15.2)	7 (7.6)	0 (0.0)	2 (2.2)	4 (4.4)
			M/M (22)	2 (9.1)	0 (0.0)	2 (9.1)	2 (9.1)	0 (0.0)	1 (4.6)
		94-103	P/P (139)	59 (42.5)	18 (13.0)	12 (8.6)	1 (0.7)	7 (5.0)	0 (0.0)
			M/M (7)	3 (42.9)	1 (14.3)	1 (14.3)	1 (14.3)	1 (14.3)	0 (0.0)
		129-146	P/P (38)	19 (50.0)	9 (23.7)	7 (18.4)	4 (10.5)	2 (5.3)	4 (10.5)
			M/M (5)	3 (60.0)	2 (40.0)	1 (20.0)	1 (20.0)	0 (0.0)	0 (0.0)
		176-203	P/P (36)	5 (13.9)	2 (5.6)	1 (2.8)	2 (5.6)	0 (0.0)	3 (8.3)
			M/M (0)	n. a.	n. a.	n. a.	n. a.	n. a.	n. a.
**Longi-tudinal (180)**	-	P/P T1: 7-63	P/P T1 (90; 2 NA)	35 (39.8)	15 (17.1)	11 (12.5)	4 (4.6)	2 (2.2)	3 (3.4)
		P/P T2: 121-203	P/P T2 (90)	33 (36.7)	12 (13.3)	8 (8.9)	3 (3.3)	1 (1.1)	9 (10.0)

Different vaccines and combinations are abbreviated as follows: M/M (two-dose mRNA-1273), P/P (two-dose BNT162b2), A/A (two-dose AZD1222), A/M (first dose AZD1222, second dose mRNA-1273), A/P (first dose AZD1222, second dose BNT162b2) and J (one-dose Ad26.CoV2.S). The time points sample cohort contains only homologous BNT162b2 and mRNA-1273 samples. The longitudinal sample cohort contains only paired homologous BNT162b2 taken at time 1 (T1) or 2 (T2).
***** based on self-reported positive PCR/antigen test result at study center visit and/or MULTICOV-AB nucleocapsid IgG S/CO ratio above 1

Supplementary Table 2. MULTICOV-AB antigen panel.

Virus	Antigen	Manufacturer	Product number
SARS-CoV-2	Spike Trimer	NMI	-
SARS-CoV-2	RBD B.1 (wild-type)	NMI	-
SARS-CoV-2	Nucleocapsid	Aalto	6404-b
SARS-CoV-2	RBD B.1.1.7 (Alpha)	NMI	
SARS-CoV-2	RBD B.1.351 (Beta)	NMI	
SARS-CoV-2	RBD P.3 (Gamma)	NMI	
SARS-CoV-2	RBD B.1.617.2 (Delta)	NMI	
hCoV-OC43	S1 domain	NMI	-
hCoV-OC43	Nucleocapsid	NMI	-
hCoV-HKU1	S1 domain	NMI	-
hCoV-HKU1	Nucleocapsid	NMI	-
hCoV-NL63	S1 domain	NMI	-
hCoV-NL63	Nucleocapsid	NMI	-
hCoV-229E	S1 domain	NMI	-
hCoV-229E	Nucleocapsid	NMI	-

List of antigens included in MULTICOV-AB as well as their manufacturer and, if applicable, their product number.

Supplementary Table 3. ACE2-RBD competition antigen panel.

Virus	Antigen	Manufacturer	Amino acid exchanges in RBD
SARS-CoV-2	RBD B.1 (wild-type)	NMI	-
SARS-CoV-2	RBD B.1.1.7 (Alpha)	NMI	N501Y
SARS-CoV-2	RBD B.1.351 (Beta)	NMI	N501Y, E484K, K417N
SARS-CoV-2	RBD P.1 (Gamma)	NMI	N501Y, E484K, K417T
SARS-CoV-2	RBD B.1.617.2 (Delta)	NMI	T478K, L452R

List of antigens included in ACE2-RBD competition assay as well as their manufacturer, and the mutations covered within the RBD.

Supplementary Table 4. Impact of confounders on ACE2 inhibition response towards SARS-CoV-2 wild-type and VoC RBDs.

**A. WT ACE2 binding inhibition**, logit scale	**P/P, n=734**		**M/M, n=272**		**A/P, n=114**		**A/A, n=228**	
	Est (sd)	p-value	Est (sd)	p-value	Est (sd)	p-value	Est (sd)	p-value
Intercept	3.50 (0.231)	---	3.39 (0.330)	---	3.07 (0.538)	---	-0.52 (0.496)	---
Male	-0.25 (0.103)	0.014	-0.05 (0.149)	0.752	-0.33(0.267)	0.218	0.30(0.152)	0.050
Age (per 1 year)	-0.03 (0.003)	<0.001	0.00 (0.005)	0.431	-0.01 (0.010)	0.251	0.00(0.008)	0.960
Days post-vaccination (ΔT**)**								
7-27	Ref.		Ref.		Ref.		Ref.	
28-65	-0.69 (0.104)	<0.001	-0.97 (0.169)	<0.001	-0.74 (0.260)	0.005	-0.10(0.188)	0.584
Comorbidities								
Cardiovascular	-0.12(0.158)	0.454	-0.54 (0.309)	0.083	-0.03(0.424)	0.937	0.02 (0.241)	0.943
Hypertension	0.19 (0.119)	0.109	-0.13 (0.180)	0.480	-0.08(0.301)	0.789	0.11(0.161)	0.485
Diabetes	-0.04 (0.185)	0.808	0.58(0.386)	0.136	0.84(0.501)	0.095	-0.15(0.242)	0.547
Lung disease*	-0.03(0.187)	0.853	0.53(0.324)	0.104	-0.87(0.513)	0.091	-0.11(0.349)	0.758
Cancer or Immunosuppression*	-0.26(0.192)	0.176	0.29(0.362)	0.418	0.22(0.508)	0.662	-0.61(0.368)	0.099

**B. Alpha ACE2 binding inhibition**, logit scale	**P/P, n=734**		**M/M, n=272**		**A/P, n=114**		**A/A, n=228**	
	Est (sd)	p-value	Est (sd)	p-value	Est (sd)	p-value	Est (sd)	p-value
Intercept	2.50(0.211)	---	2.64(0.301)	---	2.26(0.465)	---	-0.77(0.420)	---
Male	-0.22(0.083)	0.008	-0.02(0.130)	0.862	-0.27(0.226)	0.228	0.24(0.126)	0.060
Age (per 1 year)	-0.02(0.003)	<0.001	-0.01(0.004)	0.218	-0.01(0.009)	0.270	0.00(0.006)	0.826
Days post-vaccination (ΔT)								
7-27	Ref.		Ref.		Ref.		Ref.	
28-65	-0.56(0.083)	<0.001	-0.89(0.147)	<0.001	-0.58(0.219)	0.008	-.03(0.155)	0.842
Comorbidities								
Cardiovascular	-0.10(0.125)	0.434	-0.42(0.266)	0.112	-0.02(0.361)	0.961	0.04(0.202)	0.842
Hypertension	0.16(0.095)	0.087	-0.10(0.156)	0.524	-0.06(0.255)	0.800	0.05(0.134)	0.715
Diabetes	-0.03(0.149)	0.828	0.41(0.332)	0.216	0.81(0.425)	0.057	-0.05(0.204)	0.815
Lung disease*	-0.03(0.149)	0.851	0.40(0.281)	0.156	-0.78(0.433)	0.073	-0.08(0.288)	0.789
Cancer or Immunosuppression*	-0.15(0.153)	0.343	0.21(0.314)	0.510	0.15(0.428)	0.724	-0.45(0.302)	0.139

**C. Beta ACE2 binding inhibition**, logit scale	**P/P, n=734**		**M/M, n=272**		**A/P, n=114**		**A/A, n=228**	
	Est (sd)	p-value	Est (sd)	p-value	Est (sd)	p-value	Est (sd)	p-value
Intercept	1.27(0.259)	---	1.55(0.309)	---	1.12(0.420)	---	-1.18(0.432)	---
Male	-0.19(0.070)	0.006	-0.08(0.105)	0.448	-0.27(0.180)	0.131	0.11(0.116)	0.337
Age (per 1 year)	-0.02(0.002)	<0.001	0.00(0.004)	0.170	-0.01(0.007)	0.268	0.00(0.006)	0.584
Days post-vaccination (ΔT)								
7-27	Ref.		Ref.		Ref.		Ref.	
28-65	-0.44(0.070)	<0.001	-0.86(0.119)	<0.001	-0.59(0.175)	0.001	0.07(0.144)	0.645
Comorbidities								
Cardiovascular	0.00(0.106)	0.971	-0.37(0.216)	0.088	-0.04(0.294)	0.899	0.06(0.181)	0.741
Hypertension	0.08(0.081)	0.306	-0.12(0.126)	0.323	-0.02(0.205)	0.923	0.01(0.122)	0.940
Diabetes	0.01(0.124)	0.917	0.24(0.270)	0.380	0.48(0.345)	0.165	0.09(0.180)	0.636
Lung disease*	-0.08(0.126)	0.520	0.39(0.227)	0.087	-0.54(0.346)	0.117	0.10(0.268)	0.705
Cancer or Immunosuppression*	-0.19(0.130)	0.155	0.13(0.254)	0.606	0.27(0.340)	0.426	-0.40(0.286)	0.163

**D. Gamma ACE2 binding inhibition**, logit scale	**P/P, n=734**		**M/M, n=272**		**A/P, n=114**		**A/A, n=228**	
	Est (sd)	p-value	Est (sd)	p-value	Est (sd)	p-value	Est (sd)	p-value
Intercept	1.31(0.169)	---	1.64(0.250)	---	1.24(0.377)	---	-1.05(0.313)	---
Male	-0.20(0.067)	0.003	-0.06(0.109)	0.612	-0.36(0.183)	0.049	0.13(0.092)	0.165
Age (per 1 year)	-0.02(0.002)	<0.001	0.00(0.004)	0.222	-0.01(0.007)	0.183	0.00(0.005)	0.382
Days post-vaccination (ΔT)								
7-27	Ref.		Ref.		Ref.		Ref.	
28-65	-0.46(0.068)	<0.001	-0.91(0.124)	<0.001	-0.52(0.177)	0.004	-0.03(0.114)	0.769
Comorbidities								
Cardiovascular	-0.06(0.101)	0.556	-0.41(0.225)	0.069	-0.05(0.299)	0.862	0.13(0.145)	0.352
Hypertension	0.14(0.078)	0.063	-0.08(0.132)	0.548	0.04(0.208)	0.862	0.07(0.097)	0.496
Diabetes	-0.01(0.118)	0.960	0.21(0.281)	0.463	0.53(0.351)	0.134	0.03(0.145)	0.821
Lung disease*	-0.04(0.121)	0.770	0.27(0.237)	0.252	-0.62(0.351)	0.076	-0.13(0.212)	0.554
Cancer or Immunosuppression*	-0.17(0.125)	0.178	0.12(0.265)	0.654	0.17(0.344)	0.626	-0.45(0.225)	0.047

**E. Delta ACE2 binding inhibition**, logit scale	**P/P, n=734**		**M/M, n=272**		**A/P, n=114**		**A/A, n=228**	
	Est (sd)	p-value	Est (sd)	p-value	Est (sd)	p-value	Est (sd)	p-value
Intercept	2.34(0.218)	---	2.53(0.312)	---	2.16(0.468)	---	-1.10(0.430)	---
Male	-0.21(0.085)	0.013	-0.03(0.135)	0.830	-0.34(0.226)	0.132	0.20(0.128)	0.128
Age (per 1 year)	-0.02(0.003)	<0.001	0.00(0.004)	0.349	-0.01(0.009)	0.203	0.00(0.007)	0.698
Days post-vaccination (ΔT)								
7-27	Ref.		Ref.		Ref.		Ref.	
28-65	-0.55(0.086)	<0.001	-0.99(0.152)	<0.001	-0.70(0.220)	0.001	0.01(0.159)	0.938
Comorbidities								
Cardiovascular	-0.10(0.130)	0.445	-0.52(0.276)	0.058	0.12(0.365)	0.744	0.09(0.203)	0.641
Hypertension	0.15(0.099)	0.125	-0.11(0.162)	0.501	0.01(0.256)	0.957	-0.04(0.136)	0.762
Diabetes	-0.01(0.152)	0.969	0.42(0.345)	0.227	0.68(0.429)	0.116	-0.01(0.204)	0.953
Lung disease*	-0.02(0.155)	0.920	0.39(0.292)	0.182	-0.67(0.434)	0.125	-0.15(0.295)	0.604
Cancer or Immunosuppression*	-0.18(0.159)	0.261	0.17(0.326)	0.603	0.06(0.428)	0.888	-0.50(0.311)	0.111

ACE2 binding inhibition towards SARS-CoV-2 wild-type (A) and Alpha (B), Beta (C), Gamma (D), Delta (E) VoC in the mix and match sample cohort was modelled to assess the role of confounders (see methods for model details). Four samples (P/P) had missing information on comorbidities and were excluded from the analysis. Est=estimate, sd=standard deviation. Categories denoted with * were merged in the sensitivity analysis including interaction terms with time post-vaccination due to limited sample sizes (selected results reported in the main text). Different vaccines and combinations are abbreviated as follows: M/M (two-dose mRNA-1273), P/P (two-dose BNT162b2), A/A (two-dose AZD1222), A/M (first dose AZD1222, second dose mRNA-1273), A/P (first dose AZD1222, second dose BNT162b2) and J (one-dose Ad26.CoV2.S).

.

Supplementary Table 5. Statistical comparison of antibody titres between vaccination schemes.

**Comparison X**	**vs. Y**	**Full-length Spike trimer p'' (p-value)**	**RBD p'' (p-value)**	**S1 p'' (p-value)**	**S2 p'' (p-value)**
J	M/M	0.91 (< 0.001) *	0.93 (<0.001) *	0.96 (<0.001) *	0.86 (< 0.001) *
	P/P	0.89 (<0.001) *	0.90 (<0.001) *	0.92 (<0.001) *	0.79 (< 0.001) *
	A/M	0.89 (<0.001) *	0.91 (<0.001) *	0.97 (<0.001) *	0.93 (<0.001) *
	A/P	0.90 (<0.001) *	0.92 (<0.001) *	0.95 (<0.001) *	0.88 (<0.001) *
	A/A	0.63 (<0.080)	0.74 (<0.001) *	0.86 (<0.001) *	0.81 (<0.001) *
A/A	M/M	0.96 (<0.001) *	0.94 (<0.001) *	0.93 (<0.001) *	0.57 (0.013) *
	P/P	0.91 (<0.001) *	0.87 (<0.001) *	0.83 (<0.001) *	0.39 (<0.001) *
	A/M	0.93 (<0.001) *	0.91 (<0.001) *	0.93 (<0.001) *	0.84 (< 0.001) *
	A/P	0.93 (<0.001) *	0.89 (<0.001) *	0.91 (<0.001) *	0.66 (< 0.001) *
A/P	M/M	0.60 (0.002) *	0.64 (<0.001) *	0.55 (0.114)	0.38 (< 0.001) *
	P/P	0.42 (0.012)	0.49 (0.608)	0.34 (<0.001) *	0.21 (< 0.001) *
	A/M	0.59 (0.204)	0.59 (0.155)	0.69 (0.004) *	0.77 (<0.001) *
A/M	M/M	0.50 (0.982)	0.53 (0.623)	0.33 (0.014) *	0.16 (<0.001) *
	P/P	0.33 (0.014)	0.40 (0.085)	0.18 (<0.001)*	0.09 (<0.001) *
P/P	M/M	0.69 (<0.001) *	0.64 (<0.001) *	0.72 (<0.001) *	0.71 (<0.001) *

Estimated probability (p'') that a random individual under one vaccination scheme in the mix and match sample cohort, has a larger titre than a random individual under another vaccination scheme. Two-sided generalized Wilcoxon also referred to as Brunner-Munzel test was used to determine statistical significance with Bonferroni-Holm's adjustment for multiple testing. For a comparison of a scheme X with a scheme Y, a significant p'' larger than ½ means that the vaccination-induced titres under Y tend to be higher and a significant p'' smaller than ½ means that the vaccination-induced titres under X tend to be higher. Different vaccines and combinations are abbreviated as follows: M/M (two-dose mRNA-1273), P/P (two-dose BNT162b2), A/A (two-dose AZD1222), A/M (first dose AZD1222, second dose mRNA-1273), A/P (first dose AZD1222, second dose BNT162b2) and J (one-dose Ad26.CoV2.S).

Supplementary Table 6. Statistical comparison of RBD antibody titres and wild-type ACE2 binding inhibition in recovered-vaccinated and SARS-CoV-2 naïve-vaccinated individuals for different vaccination schemes.

	**M/M p" (p-value)**	**P/P p" (p-value)**	**A/M p" (p-value)**	**A/P p" (p-value)**	**A/A p" (p-value)**	**J p" (p-value)**
RBD	0.66 (0.074)	0.79 (<0.001)	-- (only 1 convalescent)	0.79 (<0.001)	0.94 (<0.001)	0.98 (<0.001)
AC2 binding inhibition against WT	0.74 (0.016)	0.85 (<0.001)	-- (only 1 convalescent)	0.84 (0.004)	0.97 (<0.001)	0.98 (<0.001)

Estimated probability (p'') that a random recovered-vaccinated individual has a larger titre than a random SARS-CoV-2 naïve vaccinated individual under the respective vaccination scheme. Statistical analysis was determined by two-sided generalized Wilcoxon/Brunner-Munzel test. A significant p'' larger than ½ means that the vaccination-induced titres tend to be higher in recovered-vaccinated individuals. Different vaccines and combinations are abbreviated as follows: M/M (two-dose mRNA-1273), P/P (two-dose BNT162b2), A/A (two-dose AZD1222), A/M (first dose AZD1222, second dose mRNA-1273), A/P (first dose AZD1222, second dose BNT162b2) and J (one-dose Ad26.CoV2.S).

Supplementary Table 7. Antibody titres and ACE2 binding inhibition up to 7 months after SARS-CoV-2 mRNA vaccination.

Sample cohort (n)	ΔT range in days post-vaccination	Vaccine (n)	RBD_{wt} IgG signal (95% CI)	(%) ACE2 binding inhibition (95% CI)	RBD_{wt} IgG signal (IQR)	(%) ACE2 binding inhibition (IQR)
Time points (597)	5-12	P/P(107)	24.88 (20.02-27.10)	78.73 (64.37-87.92)	24.88 (15.09-31.17)	78.73 (52.27-94.27)
		M/M (40)	26.76 (21.38-30.47)	89.03 (65.66-97.16)	26.76 (12.24-31.69	89.03 (41.06-97.95)
	26-30	P/P (103)	25.60 (23.15-27.79)	83.34 (78.04-0.86.00)	25.60 (18.76-30.87)	83.34 (63.63-93.37)
		M/M (8)	29.45 (21.01-34.13)	95.75 (60.74-96.97)	29.45 (26.20-32.42)	95.75 (77.52-96.82)
	54-58	P/P (92)	19.96 (18.22-22.73)	70.59 (62.66-75.07)	19.96 (14.00-26.37)	70.59 (58.54-83.18)
		M/M (22)	26.52 (23.56-30.01)	88.22 (78.90-94.10)	26.52 (23.54-30.20)	88.22 (77.92-94.32)
	94-103	P/P (139)	15.07 (13.97-16.77)	56.22 (50.18-61.20)	15.07 (10.20-20.70)	56.22 (39.97-69.67)
		M/M (7)	18.85 (11.67-31.61)	75.94 (43.40-96.95)	18.85 (14.18-28.24)	75.94 (55.40-90.74)
	129-146	P/P (38)	8.67 (7.24-10.27)	33.82 (27.95-39.87)	8.67 (5.48-12.68)	33.82 (25.52-50.97)
		M/M (5)	13.51 (n. a)	48.16 (n. a.)	13.51 (4.78-19.64)	48.16 (17.95-68.86)
	176-203	P/P (36)	7.97 (5.98-10.03)	30.44 (23.38-42.16)	7.97 (5.51-11.36)	30.44 (20.50-48.77)
		M/M (0)	n. a.	n. a.	n. a.	n. a.

Median values with 95% CI and IQR are shown for normalised RBD_{wt} IgG signal or ACE2 binding inhibition. Data is graphically displayed in Figure 6. Different vaccines are abbreviated as follows: M/M – two-dose mRNA-1273. P/P – two-dose BNT162b2.
